# Multiplexed Profiling of Extracellular Vesicles for Biomarker Development

**DOI:** 10.1007/s40820-021-00753-w

**Published:** 2021-12-02

**Authors:** Cheng Jiang, Ying Fu, Guozhen Liu, Bowen Shu, Jason Davis, George K. Tofaris

**Affiliations:** 1 Nuffield Department of Clinical Neurosciences, New Biochemistry Building, University of Oxford, Oxford, OX1 3QU UK; 2 Department of Chemistry, University of Oxford, Oxford, OX1 3QZ UK; 3 School of Life and Health Sciences, The Chinese University of Hong Kong, Shenzhen, 518172 People’s Republic of China; 4 Dermatology Hospital, Southern Medical University, Guangzhou, 510091 People’s Republic of China; 5 Kavli Institute for Nanoscience Discovery, New Biochemistry Building, University of Oxford, Oxford, UK

**Keywords:** Multiplexed profiling, Extracellular vesicles, Exosomes, Liquid biopsy, Point-of-care, Biomarker

## Abstract

Extracellular vesicles (EVs) are cell-derived membranous particles that play a crucial role in molecular trafficking, intercellular transport and the egress of unwanted proteins. They have been implicated in many diseases including cancer and neurodegeneration. EVs are detected in all bodily fluids, and their protein and nucleic acid content offers a means of assessing the status of the cells from which they originated. As such, they provide opportunities in biomarker discovery for diagnosis, prognosis or the stratification of diseases as well as an objective monitoring of therapies. The simultaneous assaying of multiple EV-derived markers will be required for an impactful practical application, and multiplexing platforms have evolved with the potential to achieve this. Herein, we provide a comprehensive overview of the currently available multiplexing platforms for EV analysis, with a primary focus on miniaturized and integrated devices that offer potential step changes in analytical power, throughput and consistency.

## Highlights

- Extracellular vesicle (EV) multiplexing involves chemical, physical spatial, biological, or nanoparticle-based strategies.
- Multiplexing in EV biomarker development includes high-throughput screening as well as point-of-care testing platforms which to date have been applied mainly to EV surface proteins or internal cargo miRNAs.
- Multiplexed measurements at single-EV resolution are likely to revolutionize the applicability of EV analytes as biomarkers in complex and heterogeneous diseases.

## Graphical abstract

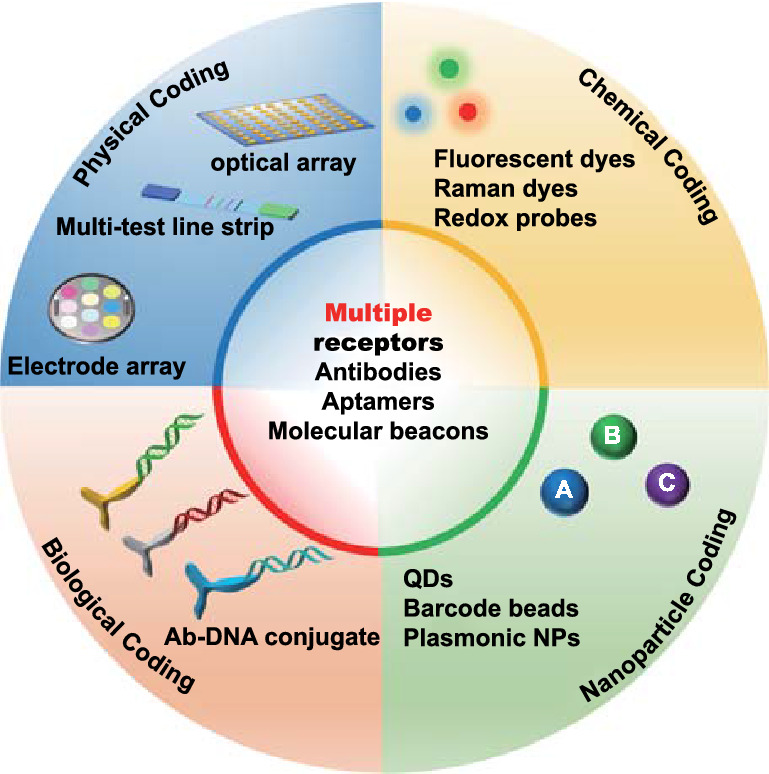

## Introduction

**Fig. 1:**
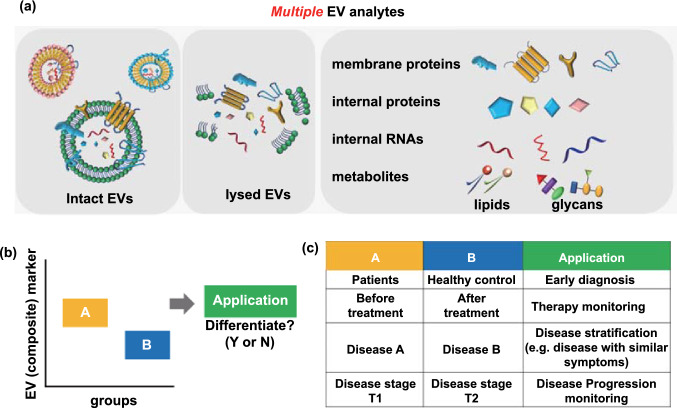
Potential clinical applications of composite EV biomarkers. **a** The molecular profiling of EVs may be based on proteomics (e.g. membrane proteins and internal proteins), RNAs or metabolites (e.g. lipids and glycans). **b** The multiplexed analysis of EV components can generate a box plot representation of expression levels in A and B groups as defined in **c**. **c** Potential clinical applications of EV markers

Circulating extracellular vesicles (EVs) are a heterogeneous group of vesicles secreted by a variety of cell types, present in most bodily fluids (e.g. blood, saliva, urine, ascites, cerebrospinal fluid) and thought to function in intercellular communication by transferring cargo molecules from donor to recipient cells or facilitating the jettison of unwanted molecules to the cell exterior [1, 2]. A large fraction of EVs consists of exosomes (30–150 nm in diameter), which are endosome-derived EV or ectosomes (0.1–1.0 µm) derived from the plasma membrane. EVs carry parental cell-derived fingerprint proteins and RNAs but can also envelop disease-associated proteins and microRNAs [3, 4]. As such they are diagnostically relevant to cancers [5–10], neurodegenerative diseases [11–15], and cardiovascular disease [16, 17]. The potential applications of EV analysis in diagnosis and prognosis, are, then, substantial (Fig. 1a), especially if multiple targets can be screened simultaneously [18–20]. In this regard, multiplexed profiling has the advantage of increasing diagnostic accuracy and reducing sample volume and assay time as well as variability arising from the repeated processing of multiple sample aliquots for serial singleplexing. Due to the heterogeneity and complexity of human diseases, it is becoming clear that a single biomarker is unlikely to offer an accurate estimate of either a pathological state or phase. An efficient assessment of combinatorial markers has emerged as a promising way of measuring diseases with complex multifactorial aetiologies, and ultimately developing objective readouts for prognosis, stratification or therapy monitoring (Fig. 1b, c) [21–23].

Prior to any downstream analysis, EVs need to be isolated from complex biofluids. This can be achieved by utilizing their physical properties (e.g. size, density, surface charge) or surface markers as reviewed elsewhere [24–26]. Here we aim to provide a comprehensive overview of the fundamental strategies used to build multiplexing platforms for intact EVs and EV-derived proteins, RNAs, metabolites, and their application to clinical samples. We have also included platforms that have not yet been applied to EVs but could be adapted for such purpose and discuss how this technology may evolve in the future.

## Overview of EV Multiplexed Profiling Strategies

**Fig. 2:**
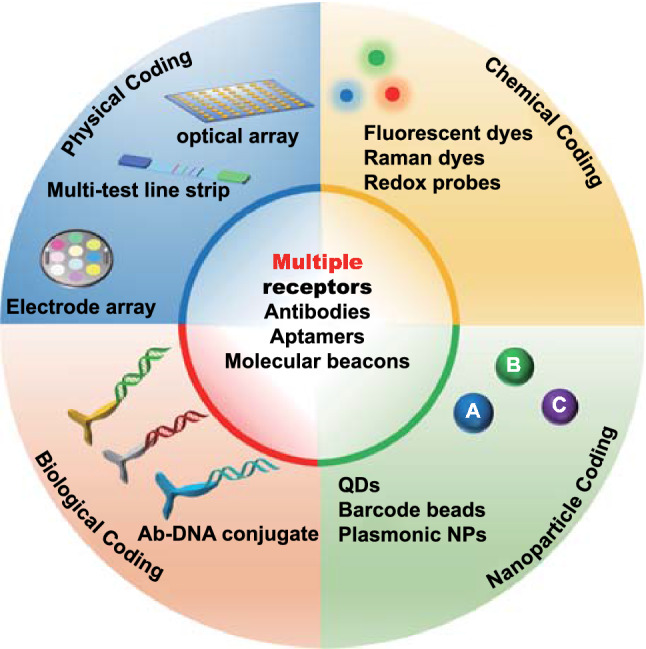
Main external-coding strategies for EV profiling. Multiplexing is typically based on the combination of multiple receptors with one of the four coding strategies. Depending on how the analyte signal is generated and transduced, multiple external codes generated by chemical reporter labelling, physical spatial coding, biological coding, or nanoparticle coding, can be used in association with multiple receptors (QDs = quantum dots, NP = nanoparticles)

The multiplexed profiling of EVs should be considered as the capability of one detection platform to assay multiple EV-derived analytes, typically proteins, RNAs and metabolites in a short time scale. The innate requirement for each analyte to generate a distinguishable signal can be achieved by two generic strategies, i.e. an intrinsic fingerprint-based strategy (internal coding) or an external coding-based strategy (external coding). The former strategy employs native properties such as charge/mass (m/z) based mass spectroscopy, as reviewed elsewhere [27, 28]. The latter, as shown in Fig. 2, typically involves the use of multiple receptors (e.g. antibodies, aptamers, molecular beacons*,* or locked nucleic acids, LNA) as recognition components, in association with multiple reporters, e.g. chemical labels (optical dyes, redox probes), DNA oligonucleotides, or nanoparticle tags [29], for distinct signal generation derived from the binding events of multiple EV analytes and/or physically isolated regions of a solid interface such as those within multi-spot optical arrays or electrochemical arrays (i.e., position coding). Accordingly, the EV multiplexed profiling strategies can be divided into four main categories, chemical-, physical-, biological- or nanoparticle-based coding. In practice, this is achieved by bio-affinity induced binding events between receptor and EV analyte, potentially through the cooperation of multiple receptors, and using one of these four strategies or their combination. These will each be systematically discussed in the following sections. Generally, one receptor triggers one ‘code’ with the generation of multiple distinct codes enabling multiplexing.

## Chemical Coding

For chemical coding, a range of small chemical reporters such as redox probes, fluorescent dyes and Raman tags can be employed in cooperation with capture ligands (i.e. receptors) to generate distinguishable and specific readouts for EV components. A broad range of methods are available to facilitate this, as detailed below. In this mode, different receptors, each matched with a chemical tag, bind to specific EV analytes, with each binding event generating a distinct readout. A detection of each EV analyte, i.e. the binding event of EV analyte with the corresponding code, is thus transduced into a signal for that code.

### Raman Tags

**Fig. 3:**
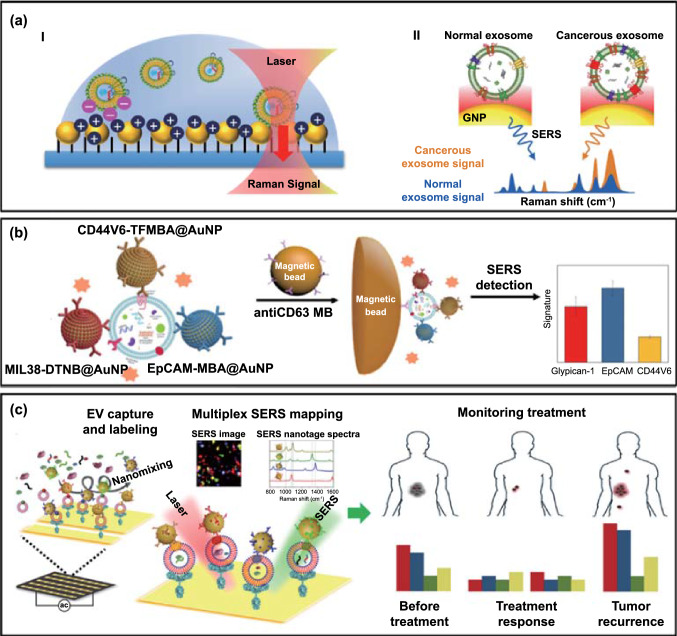
Typical SERS-based approaches for EV multiplexing. **a** An examination of multiple EV components using the bulk chemical fingerprints of immobilized EVs. Adapted with permission from Ref. [35]. Copyright 2018 American Chemical Society. **b** Schematic illustration of molecular phenotype profiling of CD63-positive EVs using SERS nanotags (antibody-Raman dye conjugate: anti-MIL38-DTNB, anti-EpCAM-MBA, and anti-CD44V6-TFMBA). Adapted with permission from Ref. [36]. Copyright 2020 American Chemical Society. TFMBA: 2,3,5,6-Tetrafluoro-4-mercaptobenzonic acid, DTNB: 5,5’-dithiobis(2-nitrobenzoic acid), MBA: 4-mercaptobenzoic acid. **c** A multiplex EV phenotype assay chip using four SERS nanotags. The phenotypic evolution can be tracked by analysing EV samples before, during, and after immunotherapy treatment, thus providing information on treatment responses and the early signs of drug resistance. Adapted with permission from Ref. [39]. Copyright 2020 American Association for the Advancement of Science (AAAS)

Raman spectroscopy permits the resolution of unique molecular fingerprints based on vibrational and rotational modes. It reflects the overall chemical bond characteristics of EV molecular fingerprints including proteins, lipids, and metabolites. The resulting spectroscopic patterns are potentially able to differentiate between the origins of EVs or disease subgroups. To bypass the natively low signal/noise ratio of Raman spectroscopy, surface-enhanced Raman spectroscopy (SERS) makes use of the strong electromagnetic fields generated at appropriately designed plasmonic substrates [30–34]. For example, a correlation between nonsmall cell lung cancer (NSCLC) cell-derived exosomes and protein markers was shown through their unique Raman scattering profiles and subsequent principal component analysis (PCA) (Fig. 3a) [35].

Such native Raman spectra reflect overall intrinsic EV chemical components, whereas specific probing of EV components using multiple Raman tag labels can be applied using a SERS system. In this way, signals from different EV analytes can be transduced using the characteristic peaks of the corresponding Raman tag. For example, Zhang et al. have very recently studied the molecular profile of CD63-positive EVs using multiple SERS tags. In this work, EVs from three different cancer types were captured with anti-CD63 magnetic beads, followed by the addition of three types of SERS nanotags (i.e. Raman reporter-detection antibody-decorated AuNPs targeting the EV membrane proteins glypican-1, EpCAM, and CD44). In this way, a composite of magnetic beads-EVs-triplex SERS nanotags was used for multiplexed detection (Fig. 3b) [36] and applied to EV proteins derived from cancer cell lines and plasma from patients with colorectal, bladder or pancreatic cancer. In similar work, aptamer-decorated magnetic beads as capture agents coupled with Raman dye conjugated aptamers@AuNPs [37] or gold nanorods (AuNRs) [38] were used as detection probes to profile specific membrane proteins from cancer-derived EVs. Wang et al. recently elaborated upon the multiplexed coding strategy by introducing four distinct SERS nanotags, i.e. AuNPs decorated with different antibodies (targeting surface proteins) and Raman reporters. Instead of using magnetic particles for EV capture, they generated anti-CD63-modified gold chips for the capture of generic EVs from plasma of melanoma patients and healthy controls (Fig. 3c) [39], followed by the addition of Raman dye-antibody@AuNPs based SERS probes against four membrane proteins. This study, in a small group of patients, demonstrated the potential of the technology in differentiating between healthy and disease states or monitoring the response to targeted therapies. It is noteworthy that, for such SERS analyses, a comprehensive consideration of EV dimensions and the size of the magnetic and gold components is necessary to avoid steric hindrance, which could affect the binding efficiency and thus the detection limit [40].

The combination of metallic substrates (planar or colloidal), receptors (e.g. antibodies, aptamers) and Raman dye-based SERS probes offers, then, one avenue for EV multiplexing, especially in the analysis of EV membrane proteins. A tuning of metallic structure can improve sensitivity such that multiplexing at single-EV level [41, 42] is possible. However, the poor reproducibility of fabrication of SERS substrate limits the clinical application of this method. A high-throughput fabrication using lithography, and the use of intrinsic EV Raman signatures for normalization may offer a solution to overcome such limitations.

### Fluorescent Dyes

**Fig. 4:**
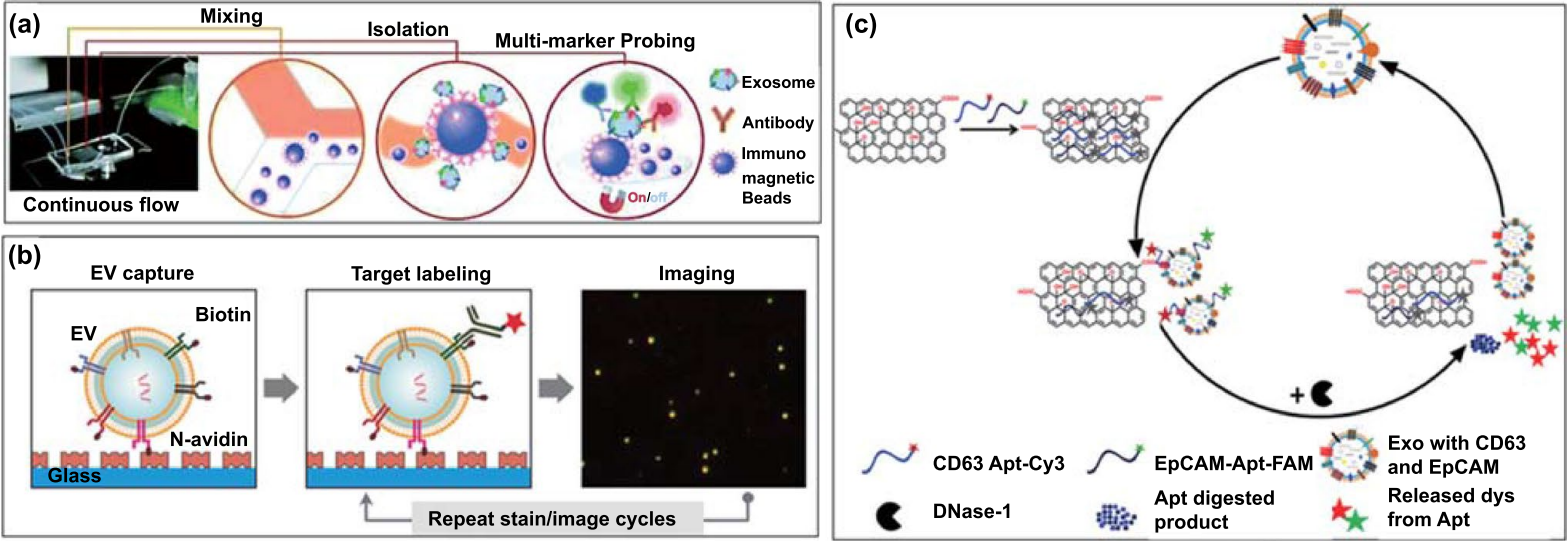
Multiplexed profiling of EV proteins using fluorescent dye-based chemical coding strategy. **a** The ExoSearch chip for continuous mixing, isolation and in situ, multiplexed detection of circulating exosomal markers CA-125, EpCAM and CD24. Reproduced with permission from Ref. [43]. Copyright 2016 Royal Society of Chemistry. **b** Multiplexed single-EV analysis by microfluidic immunofluorescence staining. Reproduced with permission from Ref. [51]. Copyright 2018 American Chemical Society. **c** The principle of an enzyme-aided fluorescence amplification based on GO-aptamer interactions for the detection of exosomal membrane proteins. Reproduced with permission from Ref. [53]. Copyright 2018 Elsevier

Besides Raman dyes, fluorescent dye-based chemical coding strategies have also been widely used for EV multiplexed profiling. This is generally achieved through the capturing of EVs on a specific receptor-modified flat or spherical interface, followed by the addition of multiple fluorescence dye-tagged ligands (e.g. antibodies, aptamers). For example, an integrated magnetic microfluidic chip with immunofluorescence has been developed by Mei and co-workers (Fig. 4a; termed the ExoSearch biochip) [43]. The magnetic bead-based microfluidic mixing-capture format offers several advantages, including rapid magnetic solid-phase isolation, flexible capture capacity, and good scalability [44–47]. In this work, plasma samples and anti-CD9 conjugated magnetic beads (MB) were injected into separate channels and, after on-chip mixing, MB-bound EVs were retained by a magnetic field followed by a multiplexed fluorescence assay using a panel of three fluorescently labelled antibodies targeting CA-125, EpCAM and CD24. The authors demonstrated high performance of the receiver operating characteristic (ROC) curve (ovarian cancer *vs* healthy control) with an area under curve (AUC) = 1 using either CA-125 or EpCAM as signature, and AUC = 0.91 for CD24. In a similar approach, Fang et al. recently developed a continuous-flow microfluidic system for the immunomagnetic capture of CD63( +) exosomes and detection of two exosomal tumour markers (EpCAM and HER2) in plasma samples using an immunofluorescence approach [48]. They demonstrated that EpCAM levels were significantly higher in exosomes from breast cancer patients compared to healthy controls. The scalability, multiplex capability, and potential for automation of the proposed magnetic microfluidics, together with larger validation cohort size and a composite marker format, may enhance its clinical translation. The integration of a chemical lysis step in another microfluidic chip enabled the quantification of a range of membrane-associated (EpCAM, α-IGF-1R, CA125, CD9, CD81, and CD63) and internal (e.g. phosphorylated type 1 insulin growth factor receptor, IGF-1R) EV proteins from two lung cancer patients, two ovarian cancer patients, and healthy controls [49].

The examples mentioned above, focused on protein quantification with bulk EV preparations that are often heterogeneous. Single-EV analyses could, in principal, offer a more granular resolution of EV subpopulations [50]. In this regard, Lee et al. have recently explored a multiplexed fluorescent imaging system for assaying markers at single-EV resolution (Fig. 4b) [51]. In their study, the EVs were biotinylated and subsequently captured on a neutravidin-coated glass within a microfluidic chamber. The immobilized EVs were immunostained using different fluorophore-conjugated antibodies targeting different EV membrane proteins, followed by fluorescence imaging. Their measurement of 11 different proteins in a single vesicle uncovered a heterogeneous marker expression profile on individual EVs derived from the same glioblastoma cell line. For example, the results revealed that only half of the EVs are CD63 positive (54%) and even fewer EVs expressed CD81 (26%) and CD9 (4.8%). In a different approach, Cavallaro et al., developed a multiple tagging strategy combining fluorescence imaging with atomic force microscopy which permits the acquirement of multidimensional information including membrane protein phenotype, size and mechanical properties simultaneously [52].

The multiplexed fluorescence profiling of EVs has been further extended to in situ liquid-phase detection using nanomaterial platforms. Want et al., for example, have explored a graphene oxide (GO) and fluorescent labelled DNA aptamers based platform for the detection of CD63 and EpCAM in colorectal cancer exosomes (Fig. 4c) [53]. Specifically, the fluorescence of fluorophore-labelled aptamers (Cy3-CD63 and FAM-EpCAM aptamers) was quenched by GO but recovered by exosome induced displacement. The DNase I enzyme was then used to digest the single-stranded DNA aptamers on the exosome surface allowing the exosomes to interact with more fluorescent aptamer probes, resulting in further increase in the fluorescence signal. Such “switch-on” and enhancement fluorescence profiling was shown to distinguish healthy from colorectal cancer patient samples using either CD63 or EpCAM. A similarly dispersed nanomaterial based approach was developed using GO with a panel of aggregation-induced emission luminogens (AIEgens) conjugated aptamers [54], or carbon nanotubes [55] or MXene Ti_3_C_2_ nanosheet [56] with different fluorescent dye labelled aptamers.

**Fig. 5:**
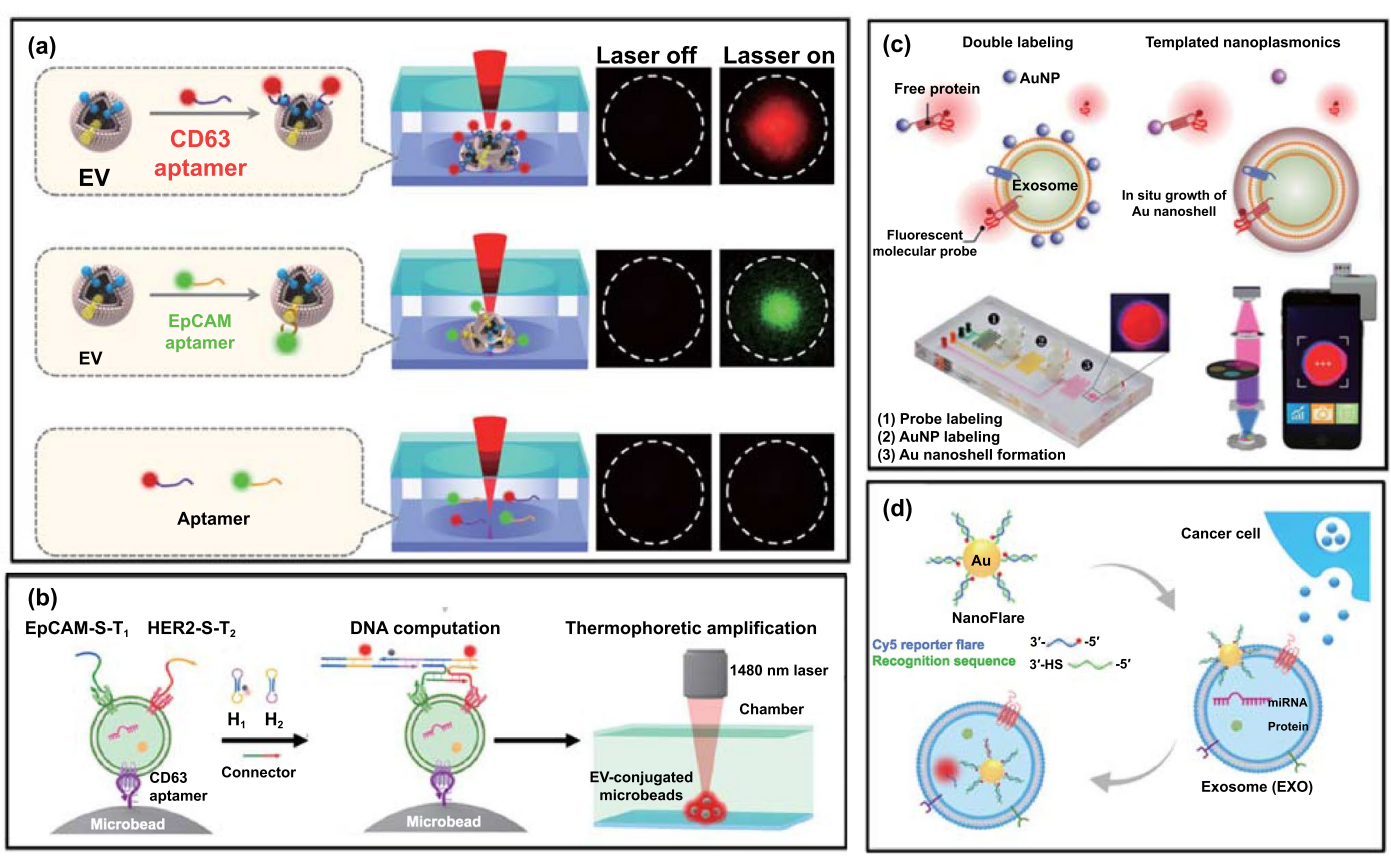
Chemical coding strategies with signal amplification for EV multiplexing. **a** An aptasensor for the thermophoretic enrichment of EVs and multiplexed profiling of their surface proteins. Reproduced with permission from Ref. [57]. Copyright 2019 Springer Nature. **b** DNA ligation system for EV membrane protein profiling using thermophoresis. Reproduced with permission from Ref. [69]. Copyright 2021 American Chemical Society. **c** Schematic of the TPEX microfluidic multiplexing platform. Exosomes were incubated with different fluorescent aptamers, either individually (singleplex) or as a mixture (multiplex), for templated plasmonics for exosome (TPEX) analysis. Reproduced with permission from Ref. [63]. Copyright 2020 American Association for the Advancement of Science (AAAS). **d** Thermophoretic sensor implemented with nanoflares for in situ detection of exosomal miRNAs. Reproduced with permission from Ref. [70]. Copyright 2020 American Chemical Society

An amplified fluorescence multiplexing methodology was also reported using a thermophoretic aptasensor (TAS) for the enrichment of EVs and the profiling of seven surface proteins (PTK7, LZH8, HER2, PSA, CA125, EpCAM and CD63) via a panel of corresponding fluorescent aptamers (Fig. 5a) [57]. The EV membrane proteins were first labelled with fluorophore conjugated aptamers, followed by laser irradiation to generate a local temperature gradient to induce a size-dependent thermophoretic accumulation of EVs larger than 30 nm. This led to a significant amplification of the fluorescent signal from aptamer bound EVs, a signal that linearly correlated with the respective protein expression levels [58]. The TAS was applied to 232 clinical serum samples from breast, liver, lung, lymph, ovary, and prostate cancer patients and healthy donors. A supervised machine learning algorithm was subsequently employed to classify different cancer types with an overall accuracy of ∼70% (Table 1). Moreover, a logistic regression analysis using these seven EV markers exhibited a superior performance to the routinely used prostate specific antigen (PSA) blood test in distinguishing prostate cancer from benign prostate disease. Taking advantage of thermophoretic accumulation, the same group further introduced a hybridization chain reaction (HCR) to achieve dual amplification for profiling multiple protein biomarkers at a single-EV level (Fig. 5b). In this study, tumour-derived EVs were first captured by CD63-aptamer-modified microbeads followed by addition of two aptamers targeting the membrane proteins EpCAM and HER2 on EVs. The HCR was initiated with the two hairpins (H1 and H2) and two connector sequences to facilitate logic operation. After HCR, a thermophoretic enrichment of EV-conjugated microbeads amplified the output signal sufficiently to discriminate HER2-positive, HER2-negative patients, and healthy controls based on the convergent thermophoresis-DNA HCR. The concept of logic gate, as utilized here, is highly dependent on multiple aptamers being in close proximity. This proximity ligation approach was additionally verified for the sensitive detection of tumour-derived exosomal PD-L1/CD63 [59], and exosomal CD63/EpCAM/MUC1 using multiple fluorophore coded aptamers [60–62].

**Table 1:** Summary of the external coding-based multiplexing profiling of EV components

Multiplexing strategy	Detection platform	EV components	EV sources	EV isolation method	Assay performance	Key findings and achievements	References
Multi-isolated channel electrode coded antibodies	Colorimetry	Surface proteins:HER2, PSA, and CD9	CCM from breast cancer and prostate cancer cell lines	Polymer precipitation	LoD = 2760 exosomes μL^−1^	Elevated exosomal HER2 were detected from HER2( +) BT-474 exosomes	[84]
Capture Ab and multiple fluorescent Ab based coding with microfluidic chip	Fluorescence	surface proteins: CA-125, EpCAM, CD24	Plasma from ovarian cancer patient and HC	On-chip immunocapture	LoD = 750 EVs μL^−1^	ROC curve (15 patients *vs* 5 HC): AUC(CA-135) = 1.000, AUC(EpCAM) = 1.000, AUC(CD24) = 0.907	[43]
Dual fluorescent aptamers	Fluorescence	surface proteins: HER2 and EpCAM	Serum from breast cancer patients	Hydrodynamic sorting on-chip	single-EV level profiling	Discrimination of breast cell lines and Stage II breast cancer patients by HER2 of microvesicles using LDA	[85]
Spatially coded Ab nanohole array	SPR	surface proteins: EpCAM, CD24, CA19-9, CLDN3, CA-125, MUC18, EGFR, HER2, CD41, CD45, D2-40	Ovarian cancer patient plasma	On-chip immunocapture	LoD = ∼3,000 exosomes (670 aM)	ROC curve (20 patients *vs* 20 HC): AUC(EpCAM) = 0.968 AUC(CD24) = 0.900, AUC(EpCAM + CD24) = 0.995	[86]
DNA-Ab conjugates based coding	Colorimetry	surface proteins: LPM1, EGFR	Plasma from NPC patients and HC	Immunocapture	LoD = 10^2^ EVs mL^−1^	ROC curve (50 stage I-II NPC patients *vs* 50 EBV-VCA-IgA-positive HC): AUC(LPM1) = 0.956 AUC(EGFR) = 0.906	[87]
Aptamer/AuNPs based coding	Colorimetry	Surface proteins: CD63, EpCAM, PDGF, PSMA, PTK7	CCM from HeLa, PC-3, Ramos, CEM cell lines	Ultracentrifugation	LoD = 3.2 μg mL^−1^	Low production of PSMA in PC‐3 cells, higher level of PTK7 from CEM exosomes than that of Ramos exosomes, absence or very low presence of PDGF in all exosomes	[88]
Aptamers based coding	Fluorescence	surface proteins: MUC1, HER, EpCAM, CD 63	CCM from five cancer cell lines of Chondrocyte, HeLa, MCF‐7, SKOV3, HepG2	Ultracentrifugation	LoD = 0.24 μg mL^−1^	Highest expression levels of MUC1, HER2, and EpCAM in MCF-7 secreted exosomes, highest expression level of CD63 in Hela-secreted exosomes	[89]
FAM-labeled aptamers based coding	Fluorescence	Surface proteins:CD63, AFP, CEA, EpCAM, PTK-7, PSMA,PDGF	CCM from cell lines of HepG2, HeLa, SGC7901 and MCF-7, MCF-10A	Ultracentrifugation	LoD = 160 EVs particles μL^−1^	Highest EpCAM level in MCF-7 exosomes, AFP displays the highest abundance on HepG2 exosomes	[56]
Ab-QDs based coding	Fluorescence	Surface proteins: CD63, CD9 and CD81	CCM from cell lines of MCF7 and MDA–MB–231	Immunpcapture on-chip	LoD = 500 exosomes μL^−1^	Drug (i.e. paclitaxel) has a modest effect on the expression level of CD9 for both cell lines	[90]
Aptamer-dye-based coding	Fluorescence	Surface proteins: PTK7, LZH8, HER2, PSA,CA125, EpCAM andCD63	Plasma from six types of cancer patients (breast, liver, lung, lymph, ovary, prostate, and HC	Ultracentrifugation	LoD = 3300 EVs μL^−1^	High accuracy (AUC = 1) of the unweighted SUM signature in cancer *vs* health discrimination	[57]
Imprinted polymer-dye based coding	Fluorescence	Surface proteins: CD9 and GGT1	CCM from PC3 prostate cell line and normal cell line, human tears	On-chip immunocapture	LoD = 6 pg mL^−1^	GGT1 can clearly separate PC3 secreted exosomes from normal exosomes	[91]
Multi-electrode spatially coded antibodies	Chronoamperometry	Surface proteins: EpCAM, CD24, CA125, HER2, MUC18 and EGFR	CCM from ovarian cancer cell lines (CaOV3, OV90, and OVCAR3), plasma from ovarian cancer patients and HC	On-chip immunocapture	LOD = 3 × 10^4^ EVs/ electrode	Both EpCAM and CD24 level in EVs are elevated in ovarian cancer patients than in HC, and both metrics showed high correlation	[92]
Spatial coding of multiple antibodies with electrode array	ECL	Internal proteins: syntenin, α-synuclein, clusterin	Serum from PD patients and HC	Immunocapture	LoD (α-synuclein) = 10 pg/mL, LoD (clusterin) = 244 pg mL^−1^), LoD (syntenin-1) = 9.7 ng mL^−1^	AUC (α-synuclein) = 0.86 for PD *vs* HC in training and validation cohorts	[93]
Spatial coding of multiple antibodies with nanohole array	SPR	Internal proteins: AKT1, HSP90, HSP70, TSG101.membrane proteins: CD63, EpCAM, EGFR	CCM from ovarian cancer cell lines (OVCAR3, OV420, CaOV3) and benign cell line (TIOSE6)	On-chip immunocapture	LoD = 2 × 10^4^ EVs μL^−1^	High expression level of HSP90 in all cell lines derived exosomes except the TIOSE6	[94]
Spatial coded antibody array	SERS	Surface proteins: EpCAM, HER2, CD44, EGFR, IGF1R, CD81, CD63, CD9	Plasma from HER2-positive breast cancer patients	On-chip immunocapture	LoD 2 × 10^3^ EVs μL^−1^	HER2-positive breast cancer patients exhibit significantly (*p* ≤ 0.01) higher level of HER2 and EpCAM than those from HC	[95]
Spatial coded antibody array within microfluidic chip	SPR	Surface proteins: CD9, CD41b, CD63, CD82, EpCAM, E-cadherin	CCM from Human hepatocellular carcinoma cell lines (MHCC97L, MHCC97H)	On-chip immunocapture	LoD = ∼4.87 × 10^7^ exosomes cm^−2^	Higher expression levels of CD9 and CD41b in exosomes from MHCC97H than in those from MHCC97L	[96]
QD-antibody conjugates-based coding	Fluorescence	Surface proteins: EpCAM, EphA2	Serum from pancreatic cancer patients and HC	Ultracentrifugation	LoD (PANC-1 EVs) = 1.9 × 10^8^ EVs	ROC curves of 20 pancreatic cancer patients *vs* 12 HC, AUC(EpCAM) = 0.92, AUC(EphA2) = 0.93	[97]
Imprinted polymer-dye-based coding	Fluorescence	5 surface proteins: CD9, CD63, GGT1, ER, Her2	Tears from breast cancer patients and HC	On-chip immunocapture	LoD = 1.2 × 10^–17^ M(1 Mol = 6.02 × 10^23^ EVs)	Breast cancer patients group can be separated from HC group using PCA (n = 5)	[98]
Parallel-channel& multiple antibodies based coding	Fluorescence	Surface proteins: CD9. CD63, CD24, EpCAM, CA125, HER2, EGFR, FRα	Plasma from cancer patients and HC	Ultracentrifugation	LoD = 21 exosomes/ μL	CD24, FRα and the SUM signature provide high diagnosis accuracy (AUC = 1) to differentiate the patient and HC groups	[99]
Multi-channel and multiple antibodies based coding	Chemiluminescence	Surface proteins: CD81, CD24, and EpCAM	Whole blood from ovarian patients and HC	On-chip immunocapture	LoD = 95 EVs μL^−1^	Expression levels of EpCAM and CD24 can be used to discriminated ovarian cancer patients and HC	[100]
Parallel electrode channel-multiple aptamers-ferrocene based coding	DPV	Surface proteins: MUC1, HER2, EpCAM, and CEA	Serum from breast cancer patients and HC	Ultracentrifugation, polymer precipitation	LoD = 946 EVs μL^−1^	Expression levels of MUCI, HER2, EpCAM, and CEA proteins on breast cancer patient-derived exosomes were all higher than those on HC-derived exosomes	[101]
Spatial coding of multiple antibodies with nanohole array	SPR	Surface proteins: EGFR, EpCAM, HER2, MUC1, GPC1, WNT2, GRP94, CD63, RAB5B, CD9	Plasma from PDAC patients and HC	On-chip immunocapture	LoD = ~ 10^3^ EVs	Combined marker panel (EGFR, EpCAM, MUC1, GPC1, and WNT2) showed 100% accuracy for the training cohort in distinguishing PDAC from HC	[102]
Multiple antibody-Raman reporter based coding	SERS	Surface proteins MCAM, LNGFR, LNGFR, ErbB3	Plasma from melanoma patient and HC	On-chip immunocapture	LoD = 100 EVs µL^−1^	Specific EV profiles involved in the development of drug resistance	[39]
Oligonucleotides-dye-based coding	FET	RNAs: miR-21 and miR-126	CCM from breast cancer cell line (MDA-MB-231)	On-chip immunocapture	LoD = 1 fM	EV extraction-lysis-miRNA isolation-miRNA detection within 5 h	[103]
Molecular beacon –dye-based coding	Fluorescence	miR-21, miR-27a and miR-375	Serum from breast cancer patients and HC	Ultracentrifugation	LoD (miR-21) = 0.116 μg mL^−1^, LoD (miR-375) = 0.287 μg mL^−1^, LoD (miR-27a) = 0.125 μg mL^−1^	All three miRNAs with higher expression can distinguish patients from HC	[104]
Multiple aptamer-dye coding	Fluorescence	RNAs: miR-375, miR-221, miR-210, miR-10b	CCM from breast cancer cell lines	Ultracentrifugation	LoD (miR-375) = 0.36 fM	miR-375, showed an accuracy of 85% for detection of oestrogen receptor-positive breast cancer at early stages (stages I, II)	[70]
Multiple molecular beacon-based coding	Fluorescence	RNAs: miR-21, miR-27a, miR-375	Plasma from Gastric cancer, HCC patients and HC	Polymer precipitation	LoD (miR-375, miR-21, miR-27a) = 10 nM	The expression ratios of miR-21 /miR-375 and miR-27 /miR-375 are higher in the tumour cell lines than in the normal cell lines	[105]
Antibody-PMP based coding	Giant magnetoresistance	20 lectins	Ascites from cancer patient and HC	Ultracentrifugation	LoD = ~ 10^4^ EVs	Ascites samples of patients with poor survival demonstrated an increased signal to distinct lectins (poor prognosis lectins: Jacalin, ConA, RCA120, PHA-E, STA, LEL, WGA, DSL, and LCA; *p* < 0.0001 for all of these lectins)	[106]

Abbreviations CCM: cell condition media, SPR: surface plasmon resonance, ROC: receiver operating characteristic curve, LDA: linear discriminant analysis, AUC: area under curve, HC: healthy control, PSA: prostate-specific antigen, EGCG: epigallocatechin-3-gallate, HCC: hepatocellular carcinoma, HC: healthy control, EpCAM: epithelial cell adhesion molecule, CEA: carcinoembryonic antigen, AFP: alpha fetoprotein, CA19-9: cancer antigen 19–9, CLDN3: Claudin 3, CA-125: Cancer antigen 125, MUC18: Mucin 18, EGFR: Epidermal growth factor receptor, HER2: Human epidermal growth factor receptor 2, ER: antioestrogen receptor, LPM1: latent membrane protein 1, NPC: nasopharyngeal carcinoma, PDGF: platelet-derived growth factor, PSMA: prostate-specific membrane antigen, PTK7: protein tyrosine kinase-7, Ramos: human acute lymphoblastic leukaemia, CEM: human acute lymphoblastic leukaemia, PC-3: human prostate cancer, DPV: differential pulse voltammetry, PDAC: pancreatic ductal adenocarcinoma, GO: graphene oxide, EXO: exosomes, MBs: molecular beacons, PD-L1: programmed death-ligand 1, FAM: 5-carboxy fluorescein, PD: Parkinson’s disease, PCA: principal component analysis, FET: field-effect transistor, PMP: polycore magnetic particles, MCSP: melanoma chondroitin sulphate proteoglycan, MCAM: melanoma cell adhesion molecule, LNGFR: low affinity nerve growth factor receptor, ErbB3: receptor tyrosine protein kinase, RCA: rolling circle amplification

In a different strategy, Wu et al. have explored an exosome-templated nanoplasmonic platform for exosome multiparametric molecular profiling (Fig. 5c) [63]. Specifically, cancer cell line conditioned media containing exosomes and free proteins were first incubated with three different fluorescent aptamers against CD63, EpCAM and MUC1 on AuNPs (~ 9.2 nm). While AuNPs remain monodisperse when associated with a protein corona, they tend to encapsulate exosomes through electrostatic interactions with the exosomal membrane. Excess unbound probes and AuNP are not removed. The AuNPs can then serve as seeds for in situ gold shell growth using exosomes as a template. Corona-coated AuNPs (or unbound AuNP) exhibit limited growth (and minimal red shift in their absorbance spectra), while AuNPs bound to the exosomal surface trigger strong localized plasmonic resonance in the infrared region. The nanoshell plasmonics locally quench the fluorescent probes only if both AuNPs and fluorophores bind on the same vesicle. The methodology thus supports an in situ analysis of EV surface proteins. The entire assay can be completed within 15 min using as little as 1 μL of sample and additionally can be integrated with a smartphone readout. Further developments with the incorporation of more advanced microfluidics, such as droplet methods [64–67] and lithography patterned array [68], would enable highly parallel processing and facilitate large-scale clinical sample handling.

The examples discussed above concern fluorescent aptamers/antibody-based fluorescence coding for EV protein profiling. MicroRNAs (miRNAs) are also abundant in EVs. They are a class of small noncoding RNAs ∼22 nucleotide long that regulate gene expression in a number of diseases, including cancer, neurodegenerative disorders, and diabetes [71] and as such they constitute a rich source of biomarkers. The main challenge in measuring exosomal miRNAs is their low abundance and instability [72].

Leveraging the versatility of thermophoretic profiling, exosomal RNA profiling can also be accomplished using RNA aptamers (Fig. 5d). To this end, Zhao et al. have introduced a nanoflare sensor comprising AuNPs (diameter of ~ 13 nm), 4 aptamers that recognise different miRNAs (targeting miR-375, miR-221, miR-210, and miR-10b), and Cy5 loaded complementary DNA probes [70]. Without contact with target miRNA, the fluorescence was quenched due to the close proximity of Cy5 to the surface of AuNPs. Upon passive uptake by exosomes, the reporter of nanoflares was displaced by the internal target miRNA, leading to an increase in fluorescence intensity. Under laser irradiation, the thermophoretic accumulation of nanoflare-treated exosomes leads to an amplified fluorescence signal upon the binding of exosomal miRNAs to nanoflares, allowing the direct measurement of exosomal miRNAs down to 0.36 fM in 0.5 μL serum samples. In this study, exosomal miR-375 showed an accuracy of 85% in detecting oestrogen receptor-positive breast cancer at early stages (stages I, II). Although this work offers a tool for EV molecular phenotyping, the requirement for an external laser source, and the potential associated damage to vesicles, requires further investigation before its translation into a point-of-care (PoC) device [70, 73].

**Fig. 6:**
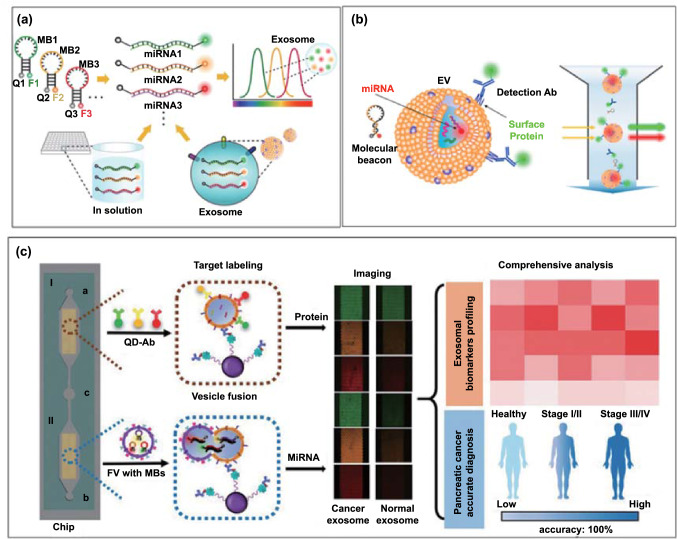
**a** Molecular beacon-based exosome internal RNA triplexing (*F*: fluorescent dye. *Q*: quencher). Reproduced with permission from Ref. [74]. Copyright 2016 Elsevier. **b** Simultaneous in situ detection of EV membrane protein and internal miRNA using dye conjugated molecular beacons and dye conjugated antibodies, respectively. Reproduced with permission from Ref. [76]. Copyright 2021 MDPI. **c** Simultaneous in situ detection of exosomal protein markers (CD81, ephrin type-A receptor 2, carbohydrate antigen 19–9) and miRNAs (miRNA-451a, miRNA-21, miRNA-10b) using QDs labelled antibody and molecular beacons using fusogenic vesicles in a microfluidic device. Reproduced with permission from Ref. [77]. Copyright 2020 John Wiley & Sons

Lee et al. developed the first molecular beacon-based assay for multiplexed detection of EV miRNAs [74]. Molecular beacons are hairpin-shaped oligonucleotide hybridization probes with a 3' fluorescent dye and 5' quencher. They serve as receptors that become fluorescent upon hybridization to a complementary RNA or DNA target sequence [75]. EV miRNAs consisting of miR-21, miR-375, and miR-27 derived from a breast cancer cell line of MCF-7 were studied (Fig. 6a). Using this approach, triple molecular beacons with unique fluorophores (FAM, Cy3, and Cy5) were successfully hybridize to three EV mRNAs (miR-21, miR-375, and miR-27) within 1 h, detecting in a single-step assay these markers in a dose-dependent manner. This in situ EV miRNA detection method avoids the need for EV lysis and RNA extraction, thus reducing the risk of RNA degradation.

To profile both EV proteins and miRNA within one configuration, Rhee and co-workers have explored the combination of fluorescent antibodies and molecular beacons for targeting membrane proteins and internal miRNAs, respectively [76]. As a proof of concept, Alexa Fluor® 488-conjugated anti-CD63 antibody and molecular beacon-21 targeting miR-21 were investigated for multiplexed biomarker detection in healthy vs cancer-derived EVs using flow cytometry (Fig. 6b). To overcome the low resolution of flow cytometry, a phospholipid-polymer-phospholipid conjugate (DSPE-PEG-DSPE) was used to induce EV clustering due to the lipid solubility of DSPE. In a more recent study, Zhou et al. proposed a vesicle membrane infusion-based strategy to obtain multi-omics information of EVs [77]. As shown in Fig. 6c, anti-CD63 polystyrene beads were used to capture exosomes in a microfluidic channel, followed by fusion with fusogenic vesicles preloaded with three molecular beacons and three antibody-conjugated quantum dots (QDs) allowing the detection of 3 internal miRNAs (miRNA-451a, miRNA-21, miRNA-10b) and 3 surface proteins (CD81, ephrin type-A receptor 2, carbohydrate antigen 19–9) through infusion-induced hybridization and immunoaffinity, respectively. The feasibility of infusion–hybridization strategy has also been extended to liposomes or cationic lipoplex nanoparticles for EV miRNA profiling in lung cancer [78–81], and human liver cancer [82]. These molecular beacon-based miRNA profiling methods are highly dependent on the integration with fluorescence microscopes; a translation to a scalable or PoC setting, mobile phones coupled with near-infrared fluorescence imaging [83] might be applied.

In summary, a number of approaches have been employed to detect multiple EV analytes (Table 1). The optical/electric/electrochemical multiplexing strategies for proteins can be readily adapted for RNAs by replacing the relevant receptors. However, fundamental issues remain: (1) the correlation between lysed EV RNA and nonlysed in situ EV RNA detection using the same platform to evaluate the uptake and capture efficiencies, (2) exploration of more generic signal amplification approaches especially when thermophoresis is not applicable. (3) Although fluorophores with different excitations can be employed for simultaneous detection, the potential for scale-up multiplexing remains due to the limited choice of chemical reporters, spectrum overlap, nonspecific adsorption and cross-talk in close proximity. For example, the overlap between the emission spectrum of FAM and the excitation spectrum of Cy5 is too small to cause Förster resonance energy transfer (FRET).

## Physical Spatial Coding

Physical spatial coding is another widely explored strategy for EV multiplexing, typically realized by positioning different receptors on an array or separated chambers targeting EV components at different spots in an addressable way (e.g. position A1, B1 represent protein A and B, respectively). The binding events of EV analytes to the receptors can be monitored using either surface sensitive label-free methods (*e.g*. surface plasmon resonance, SPR, quartz crystal microbalance, QCM, electrochemical impedance spectroscopy, EIS) or external chemical tags in a convergent physico-chemical coding fashion.

### SPR Arrays

**Fig.7:**
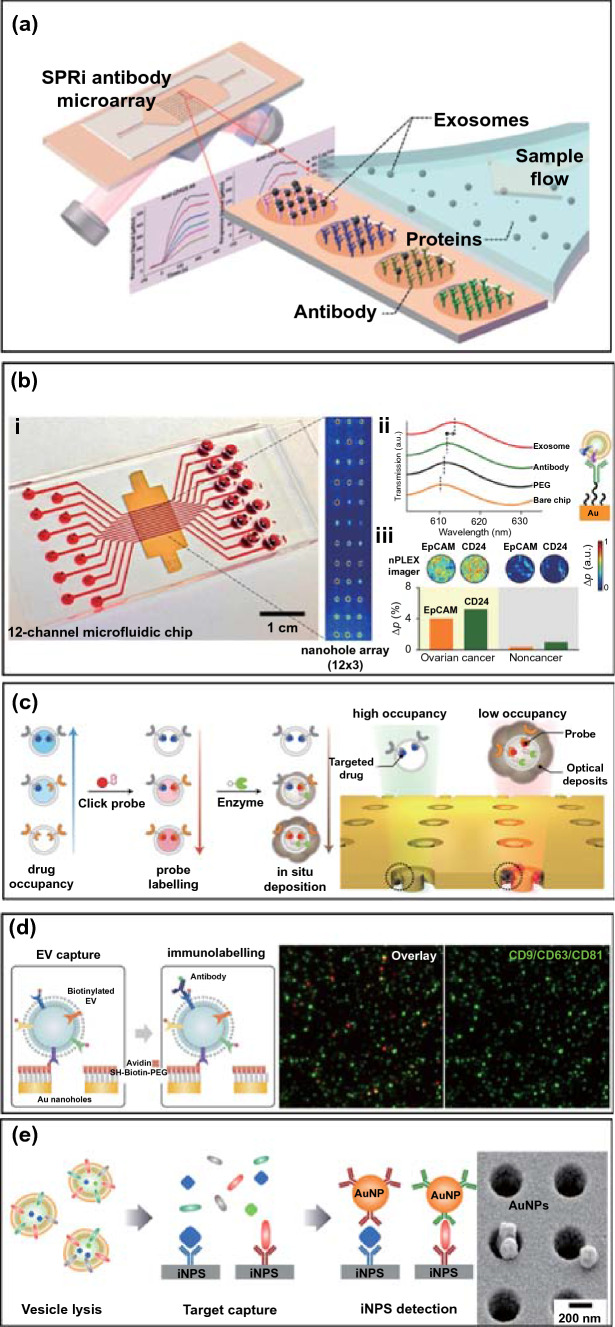
Schematic view of a physical spatial coding-based SPR platform for EV multiplexing. **a** Antibodies specific to EV transmembrane proteins were printed on the gilded gold chip, and integrated into a flow cell. Reproduced with permission from Ref. [96]. Copyright 2014 American Chemical Society. **b** nPLEX chip. (i) Integration of a multi-channel microfluidic cell for independent and parallel analyses. Transmission intensities of 12 × 3 nanohole arrays were measured simultaneously using the imaging setup. (ii) A representative schematic of changes in transmission spectra showing EV detection with nPLEX. (iii) Ascites-derived exosomes from ovarian cancer and noncancer patients were evaluated by the nPLEX sensor. Cancer EVs were captured on EpCAM and CD24-specific sensor sites with associated intensity changes in the transmitted light. Adapted with permission from Ref. [86]. Copyright 2014 Springer Nature. **c** Enzymatic amplified plasmonic sensor for EV multiplexing. Reproduced with permission from Ref. [117]. Copyright 2019 Springer Nature. **d** Surface plasmon-enhanced fluorescence biosensing for EV multiplexed profiling. Reproduced with permission from Ref. [118]. Copyright 2020 John Wiley and Sons. **e** Intravesicular nanoplasmonic system for EV multiplexing. Reproduced with permission from Ref. [94]. Copyright 2018 American Chemical Society

The surface plasmon resonance (SPR) sensing platform is exceptionally versatile for probing interfacial molecular binding events through an evaluation of the refractive index change and has been intensively investigated for highly sensitive detection of EVs [107–110]. With the integration of a charge-coupled device (CCD) camera in a spatially resolved imaging mode (SPRi), it can analyse multiple simultaneous binding events. EV multiplexed assays using an SPR sensor can usually be realized with multi-spot patterned arrays, which are spatially encoded by printing or lithography, coated with multiple EV receptors (*e.g*. antibodies) targeting specific EV components. In a typical example, Zhu et al. designed an SPR chip with an antibody microarray to simultaneously quantify multiple EV transmembrane proteins (Fig. 7a) [96]. The gold surface was printed with antibodies using a commercial microarray printer and was integrated into a microfluidic chip. Upon injection of the sample into the flow cell, EV were captured, and the associated refractive index change was monitored by the CCD camera. The SPR array has been used to detect the expression of six membrane proteins (e.g. CD9, CD41b, CD63, CD82, EpCAM, E-cadherin) on intact EV harvested from human hepatocellular carcinoma cells. The authors demonstrated that the expression of EV CD9 and CD41b was reduced after Rab27a-siRNA (i.e. small interfering RNA) treatment and increased after monensin treatment. These results demonstrated the potential of using antibody-coated microarray-based SPRi biosensors in cancer detection and drug treatment evaluation (Fig. 1c) [111].

To increase throughput capacity, Im et al. developed a plasmonic multiplexing EV platform (i.e. nPLEX) by integrating a 12 × 3 periodical nanohole gold array-based transmission SPR chip into a 12-channel PDMS chip (Fig. 7b) [86]. The chip surface was modified with mixed PEG-based SAMs (1:3, longer biotin-PEG_1000_-SH and shorter CH_3_-PEG_200_-SH) to allow antibody conjugation while resisting nonspecific fouling [112, 113]. This enabled the detection of twelve different EV membrane proteins using antibodies. Signals were further amplified by attaching anti-CD63-modified gold nanostars to captured EVs and utilizing the plasmonic coupling of these to the underlying gold surface [114–116]. The authors showed the clinical potential of this approach using ascitic fluid from 20 patients with ovarian cancer and 20 controls.

The periodic nanohole SPR array has also been employed recently to evaluate pharmacodynamics based on an examination of EV membrane protein expression after drug treatment [117]. As presented in Fig. 7c, four bio-orthogonal click probes modified with trans-cyclooctene (TCO) were designed to enable the specific targeting of three cancer proteins (EGFR, EpCAM and MUC1) and one generic EV marker (CD63). In this mode, lower drug occupancy on the EV membrane protein site allows higher orthogonal probes to engage the receptor site, leading to more enzyme (horseradish peroxidase, HRP) recruitment and in situ conversion of the soluble substrate (3,3'-diaminobenzidine) into insoluble product causing a spectral shift. This signal can be amplified due to the confined nanoring cavities where strong electromagnetic hotspots reside, so supporting a sensitive ability to monitor changes in protein composition and drug occupancy. The proposed platform was demonstrated to identify disease status and rapidly (~ 1 h) distinguish between treatment outcomes using as little as 5 μL of plasma. In another recent study, Min et al*.* explored long-range (> 100 nm) SPR-enhanced fluorescence on a periodic array instead of the localized “hotspot” surfaces (< 20 nm). This approach is more suitable for measurements with intact EVs given their dimension (30–1000 nm) [118, 119]. Four fluorescent dyes (AF488, Cy3, Cy5, and Cy5.5) were conjugated with streptavidin separately in order to code biotinylated EV membrane proteins (CD9, CD63, CD81, GAPDH, EGFR, EGFRvIII) after their capture on neutravidin-PEG layers (Fig. 7d). The PEG layer was used to prevent fluorescence quenching and to resist nonspecific adsorption [120]. This work presents a typical combination of physical and chemical coding approach powered by plasmon enhanced fluorescence (~ 10 ×).

Park et al. have extended the nanoplasmonic array (10 × 10 array) platform to measure both internal (AKT1, HSP90, HSP70, TSG101) and transmembrane proteins (CD63, EpCAM, EGFR) in lysed EVs derived from ovarian cancer cells (Fig. 7e), coupled with induced localized amplified signals generated by immunogold labelling [94]. By lysing EVs, this approach provides one way to overcome the compromise of signal enhancement in the confined “hot spot” design (i.e. detection antibody conjugated AuNPs–EV and analyte–capture antibody modified gold interface) due to steric hindrance and the dimension of intact EVs. The authors demonstrated that their sensor enabled > 10^2^–fold higher sensitivity with reduced sample input (0.5 vs. 100 µL) when compared to standard ELISA. Increased expression of HSP90 and HSP70 and decreased levels of AKT1 and TSG101 were observed in cancer cells treated with a model drug, suggesting that drug-dependent EV protein signatures may be applied to monitor therapies in patients.

In summary, the binding events of EV components to the sensing interface can be monitored by surface sensitive techniques such as SPR in a label-free format or by labelling them with one or multiple reporters, i.e. using a combination of physico-chemical coding strategies [118]. The advanced nano-/micro-fabrication-assisted SPR arrays and their integration into microfluidic chambers have been increasingly employed to facilitate better sample manipulation and miniaturization. Nanomaterial-based signal amplification approaches [110, 121, 122] can be applied to further improve the sensitivity of so developed EV sensor arrays.

### Other Spatial Coding Platforms

#### Antibody Arrays

**Fig. 8:**
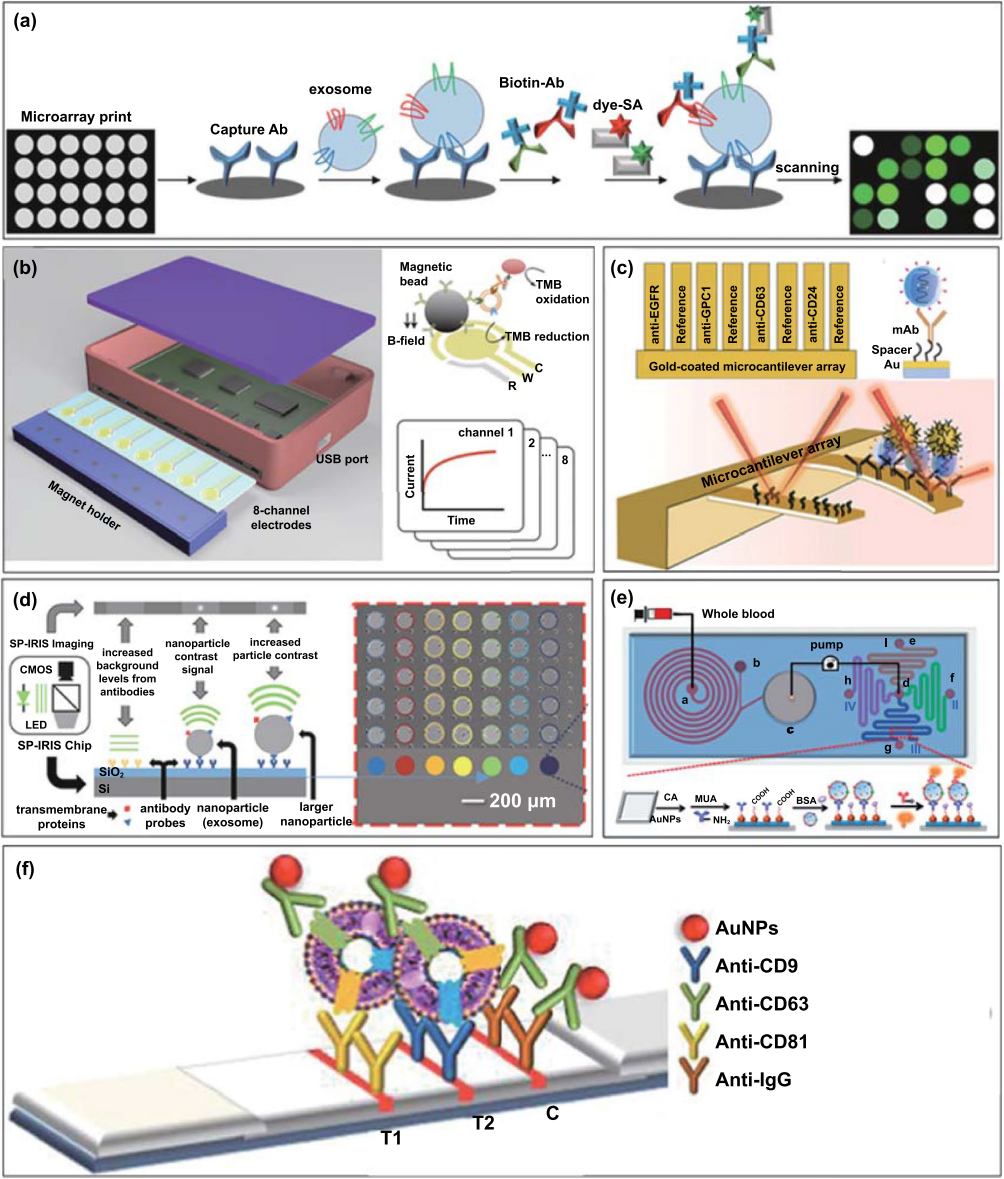
Non-SPR physical coding-based multiplexed profiling of EVs. **a** Schematic view of EV array detection of EV proteins. Adapted with permission from Ref. [123]. Copyright 2013 Taylor and Francis. **b** Integrated magnetic–electrochemical exosome (iMEX) platform. The sensor can simultaneously measure signals from eight parallel electrodes. Reproduced with permission from Ref. [92]. Copyright 2016 American Chemical Society. **c** Antibody modified cantilevers in the array with a reference control for differential detection of signal (up). Schematic of the effect of the nanoparticle mass loading on the nanomechanical deflection of the cantilever (down). Adapted with permission from Ref. [140]. Copyright 2016 Royal Society of Chemistry. **d** Schematic representation of the Single Particle Interferometric Reflectance Imaging Sensor (SP-IRIS) detection process. SP-IRIS detection principle, monochromatic LED light illuminates the sensor surface and the interferometrically enhanced nanoparticle scattering signature is captured on a CMOS camera (left). Low-magnification interferometric image showing microarray of immobilized capture probes (right). Reproduced with permission from Ref. [141]. Copyright 2016 Springer Nature. **e** Schematic diagram of an integrated microfluidic chip for plasma separation, EV detection, and molecular analysis. Reproduced with permission from Ref. [100]. Copyright 2020 American Chemical Society. **f** Multi-test line strip for profiling of EV membrane proteins. Reproduced with permission from Ref. [142]. Copyright 2017 Elsevier

Antibody array designed high-throughput multiplexing platforms, based on the immobilization of antibodies on the surface of microfluidic biochips, have been extensively applied to the analysis of EV biomarkers [123, 124]. In such configurations, an immune-based isolation of EVs is followed by a target specific immunofluorescence step. Where a panel of antibodies is spatially coded on a biochip surface, such analyses are multiplexed [125]. For example, Jørgensen et al. developed an EV microarray capable of detecting up to 60 EV proteins from 10 μL plasma (Fig. 8a) [123]. A panel of 60 captured antibodies were printed on epoxy-coated slides to recognize generic EV markers (e.g. CD9, CD63) or tumour-associated markers (e.g. EpCAM, HER2, p53). Plasma-derived EVs were captured on slides, then tagged with biotinylated anti-CD9, CD63, and CD81 antibodies prior to a readout with fluorescent streptavidin (down to detection limits of 2.5 × 10^4^ EVs per detection spot).

#### Multi-channel Electrochemical Sensors

Electrochemical detection represents another promising platform for multiplexed EV analysis owing to its intrinsic high detection sensitivity and compactness and potentially automated setup [111, 126]. Electrochemical multiplexed profiling can usually be realized by integration of multiple receptors with multi-channel electrodes (i.e. spatial position coding) and/or by cooperation with different redox probes labelled receptors, to detect the current changes as a function of the EV biomarker levels [127, 128].

For example, Jeong et al. developed a portable integrated magneto-electrochemical EV biosensor (iMEX) for multiplexed EV protein detection (Fig. 8b) [92]. The iMEX biosensor used a multi-channel (i.e. eight parallel electrodes) design offering simultaneous detection of eight markers within 1 h, with each marker requiring only 10 μL of plasma. In this work, magnetic immunobeads coated with anti-CD63 antibodies were used to enrich for CD63 positive EVs, followed by detection with antibodies against six surface proteins (see Table 1 for markers) conjugated to horseradish peroxidase (HRP) loaded magnetic beads (a chronoamperometric signal being generated). The LoD of the iMEX biosensor was 3 × 10^4^ EVs per electrode, outperforming conventional ELISA by ~ 100–fold higher sensitivity. The same group have also reported another magneto-electrochemical (multiple-electrode) sensor, termed iKEA (i.e. integrated kidney exosome analysis), to detect kidney transplant rejection. The device enabled a fast screening of T cell-derived EVs for lymphocyte-specific protein detection (CD3, CD45, CD68, CD2, HLA-ABC, CD52), showing that high levels of CD3-positive EVs identify kidney transplant rejection patients with in 91.1% of cases tested [129]. A further scale-up of this approach was reported by Tang et al., who explored an electrochemical array featuring 32 individually addressable microelectrodes multiplexed with an 8-port manifold to provide 256 sensors [130] that was successfully used to detect prostate cancer proteins in serum.

An electrode array modified with multiple receptors has also been employed for the multiplexed detection of EV miRNAs. Goda et al. have specifically reported the parallel detection of EV microRNAs (miR-143 and miR-146a) using a microelectrode array by potentiometry after reverse transcription polymerase chain reaction (RT-PCR) on EV lysates [131]. This sensing interface is based on DNA functionalized, mixed self-assembled monolayer (SAM) electrodes, comprising 5-SH-(CH_2_)_6_-DNA probe and zwitterionic sulfobetaine (SB) units as antifouling agents [113, 132]. Target hybridization events lead to changes in the interface potential which are transformed into a potentiometric signal. The platform was applied to a duplex assessment of miR-143 and miR-146a [133, 134] achieving an LOD of ~ 20 pM. Although such a platform is multi-step in nature (EV isolation-lysis-RT-PCR-detection), it is potentially readily integrated into microfluidic pre-steps and the utilized surface chemistry could be implemented in other EV analysis platforms [131, 135, 136].

#### Multi-channel Nanomechanical Sensor

Spatial coding-based EV multiplexing is also compatible with other surface sensitive detection techniques. In this regard, mechanical biosensors, e.g. microcantilevers, suspended micro-and nanochannel resonators, and quartz crystal monitoring have been utilized in bioanalysis where they can exhibit both high sensitivity and fast response [137, 138]. For example, Olcum et al. previously measured the mass of individual EVs by using a suspended nanochannel resonator (SNR) system with the ability to weigh individual nanoparticles at attogram scales [139]. Inspired by this method, Etayash et al. reported a nanomechanical cantilever array system for the simultaneous detection of multiple EV surface antigens (Fig. 8c) [140]. This nanomechanical system consisted of a gold-coated microcantilever array, on which specific EV-capturing antibodies were separately integrated. The so-generated cantilever defections generate detectable mechanical signals that scale with specific EV concentration. The use of antibody (anti-Glypican-1)-modified AuNPs to recognise and bind to pre-captured EVs further improves detection sensitivity down to ~ 200 EV mL^−1^. Despite these advances, the scalable fabrication and modification of nanomechanical biosensors remain technically challenging.

#### Interferometric Reflectance Arrays

Other label-free optical methods, such as interferometric reflectance imaging sensors (IRIS) have been used for multiplexed EV assessments. The IRIS technology is based on enhanced contrast in a scattering signal from captured nanoparticles generated on a layered substrate. This technology is widely applied to the simultaneous detection of multiple viruses in human blood [143]. Daaboul and co-workers have, for example, recently developed a surface-sensitive interferometric reflectance imaging sensors (IRIS) for the multiplexed phenotyping and digital counting of subpopulations of individual EVs captured on a microarray (Fig. 8d) using antibodies against the standard surface markers CD9, CD63 and CD81 [141]. The contrast generated is sufficient for single-EV counting with spatial location in the array encoding phenotype information. Such platforms can be readily extended to include additional EV membrane proteins by coating with corresponding receptors on different surface locations.

#### Multi-channel Microfluidics

Liu et al. designed a spinal microfluidic chip integrated with an EV detection module, enabling the direct separation and detection of EVs derived from a human ovarian cancer cell line and also plasma (Fig. 8e) [100]. The separation chip consisted of six annular microchannels that separated the plasma under inertia, reducing the interference from cell fragments and contents. The supernatant obtained after separation entered a detection module consisting of four isolated S-shaped channels, modified with capture antibodies for IgG, CD24, CD81 and EpCAM, respectively. After EV capture, HRP-labelled detection antibodies were added for downstream chemiluminescent (CL) quantification of three membrane proteins. This sensory chip was used to monitor the clinical response of ovarian cancer patients (*n* = 11) after treatment with surgical resection or chemotherapy. Levels of both CD24 and EpCAM in EVs were shown to decrease among responding patients (*n* = 8, 1–6 directed surgical resection, 8 and 9 treated with chemotherapy), indicating that the proposed device could be used to monitor response to cancer therapies.

#### Multi-test line Lateral Flow Strips

The lateral flow immunoassay (LFA) strip is a widely used assay for routine testing due to the small sample input required and low cost. In this format, pre-embedded capture antibodies bind the target analyte (i.e. at a test line) with IgG used as quality control (e.g. control line). Upon addition of the sample, AuNP decorated detection antibodies bind to the target forming a sandwich immune-complex and so generate a colorimetric signal. Blanco-Lópeza et al., for example, have introduced an EV multiplexing strip by using three antibodies targeting the corresponding membrane proteins of CD9/CD61/CD81 (Fig. 8f). Different labels (AuNPs, carbon black and magnetic nanoparticles) were examined, and AuNP-modified detection antibodies were shown to enable a sensitivity of 8.54 × 10^5^ EVs µL^−1^ using plasma samples [142]. Guo et al. have further explored the application of isotachophoresis in LFA in a paper-based isotachophoresis (ITP) format. As an electrokinetic sample-focusing technique, ITP requires a discontinuous electrolyte system comprising a leading electrolyte (LE) and a terminating electrolyte (TE) with the capability of concentrating samples by multiple orders (100−1000) of magnitude in minutes with minimal sample consumption [144]. Anti-CD44 and anti-CD63 antibodies were here used to capture cancer and generic EVs. This system performed well in human serum with target concentrations as low as 1.2–2.0 × 10^3^ EVs µL^−1^ [145].

As a highly versatile and adaptive detection platform, a wide range of engineering strategies like multiple labelling using nanomaterials, nucleic acid amplification and antifouling surface chemistry, can be employed in LFA-based EV analysis to enhance analytical performance in terms of sensitivity and selectivity [146–151]. Because of this, LFA is much cited PoC platform for EV analysis, but its utility is likely to be limited to EV surface proteins.

In summary, there is a wide range of non-SPR chip-based multiplexing platforms available, with a generally good capability of integration with functional modules like on-chip EV capture and wash. Most studies to date have focused on surface EV marker profiling, with the internal protein or RNA content not often explored. The integration of a lysis unit together with an isolation-detection module could allow a more comprehensive exploration of EV markers and accelerate biomarker discovery.

## Biological Coding

**Fig. 9:**
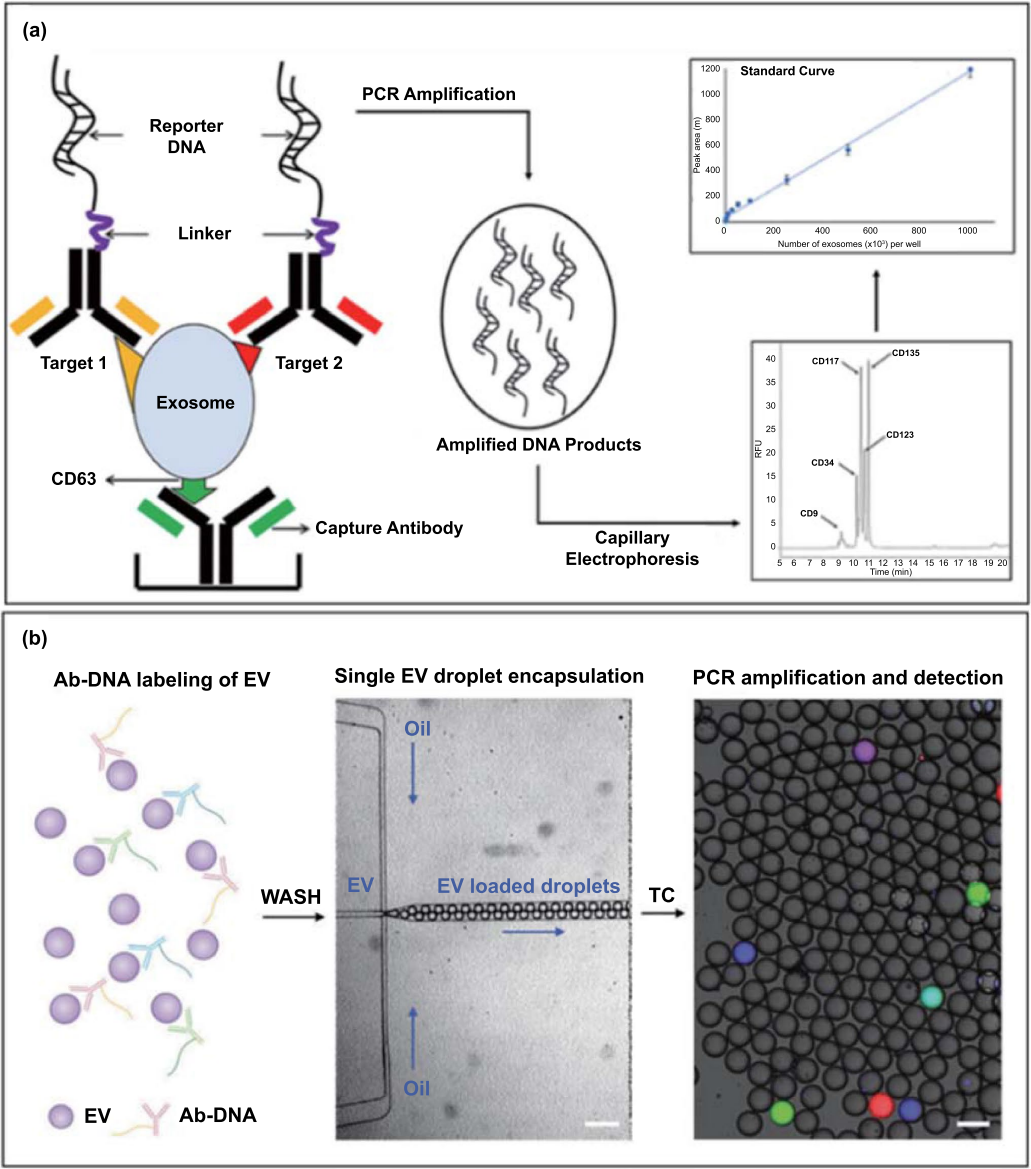
**a** Schematic assay format of immuno-PCR assisted multiplex detection of membrane proteins on EVs using capillary electrophoresis. The standard curve of the peak area in an electropherogram *vs* number of exosomes per well for multiplex immuno-PCR (upper inset). The immuno-PCR peaks for the detection of CD9, CD34, CD117, CD123, and CD135 molecules (bottom inset). Adapted with permission from Ref. [155]. Copyright 2020 American Chemical Society. **b** The convergence of antibody-DNA labelling and digital PCR for EV multiplexing. Reproduced with permission from Ref. [157]. Copyright 2020 John Wiley and Sons

In biological coding-based EV multiplexing, the EV component of interest is labelled with nucleic acids, followed by detection of these sequences, *e.g*. using chromatography or PCR, the binding event of EV analytes is converted to the detection of DNA fingerprint-based molecular tags (often with an associated significant amplification). Capillary electrophoresis (CE) can achieve high separation resolution and detection sensitivity with low sample consumption and easy maintenance [44, 152, 153]. Under optimal conditions, multiple analytes can be separated in terms of different retention time and assessed using CE owing to their different molecular weights and charges [154]. Singh et al., for example, have explored CE coupled with polymerase chain reaction-amplified immunoassay (I-PCR) for the simultaneous detection of multiple surface proteins (CD9, CD34, CD63, CD123, c-Kit/CD117, FLT-3/CD135) in EVs from acute myeloid leukaemia (AML) (Fig. 9a) [155]. In this work, EVs were first captured by anti-CD63 antibodies, followed by binding of different detection antibodies tagged with reporter DNA. Such a sandwich format combined the specificity of an immunological recognition with the exponential amplification of PCR. This method achieved the simultaneous detection of the five analytes within 12 min with a limit of detection (LOD) of 2 EVs µL^−1^ for leukaemia cell-derived EVs. A major limitation, however, concerns the poor mass separation resolution of CE. This problem can be resolved by nongel sieving CE in which entangled hydroxypropylmethylcellulose (HPMC) polymer solutions can be used instead of cross-linked gels like agarose or polyacrylamide [44, 153]. The concept of an integrated DNA amplification-capillary electrophoresis microfluidic system could enable the development of portable devices for molecular typing of EVs in other complex diseases [154, 156]. Inspired by such idea, Ko et al. have recently reported the application of antibody-DNA conjugates to label EVs, followed by stochastic microfluidic incorporation of single EVs into droplets (Fig. 9b) [157]. This was followed by in situ PCR with fluorescent reporter probes which amplify the barcode signal for subsequent detection with droplet imaging. This immune-droplet digital polymerase chain reaction (iddPCR) amplification method enabled the multiplexed profiling of EV proteins, e.g. EGFR, EPCAM, PD-L1, CD4, CD8, GZMB, TCF7 derived from cancer cell lines. This is a typical example that incorporates different coding strategies (biological and chemical coding) with amplification, to enable the detection of multiple EV membrane proteins at single-EV resolution [158–160]. It should be noted, however, that the most common formats using antibody-DNA conjugates require multiple synthesis and purification steps and a careful assessment for false-negative or false positive results (Table 2).

**Table 2:** Summary of the different EV multiplexing strategies

EV multiplexing strategies	Principle	Advantages	Limitations
Chemical	Use of multiple chemical labels to code different EV analytes	Detection of specific EV analytes using commercially available chemical labels (e.g. optical dyes, redox probes)	Limited choice of chemical labels, overlapping spectra or redox peaks
Physical	Integration of spatially isolated solid support (*e.g*. array) with multiple receptor targeting EV analytes	Compatible with high-throughput measurements in a label-free manner or specific EV marker detection using chemical labels. No potential interactions between receptors in liquid phase	Usually limited to qualitative assessment
Biological	Use of biomolecules such as DNA to code EV analytes	Dramatic signal enhancement by nucleic acid amplification, using relevant methodologies such as PCR	Multiple pre-conjugation steps and purification of receptor-biomolecule conjugates are needed
Nanoparticle	Nanoparticles with distinct optical or electrochemical properties used as labelling tags for signal detection	Wide choices of barcoded beads	Precise tuning of the optical properties of nanoparticle labels is needed

## Nanoparticle Coding

In addition to the above-discussed chemical-, physical- and biological-based EV multiplexing strategies, nanoparticle-based coding is a significant and growing option. Due to their easy synthesis and tuneable physiochemical properties, a broad range of nanoparticles bearing distinct properties (*e.g*. fluorescent-, plasmonic-, electrochemical-) have been used as labelling tags for the generation of different readouts in multiplex EV profiling. Nanoparticle coding, which can utilize numerous types of nanoparticles for signal generation to present EV analytes. This approach can be considered as an extension of the chemical tag-based coding approach.

### Fluorescent Barcoded Beads

**Fig. 10:**
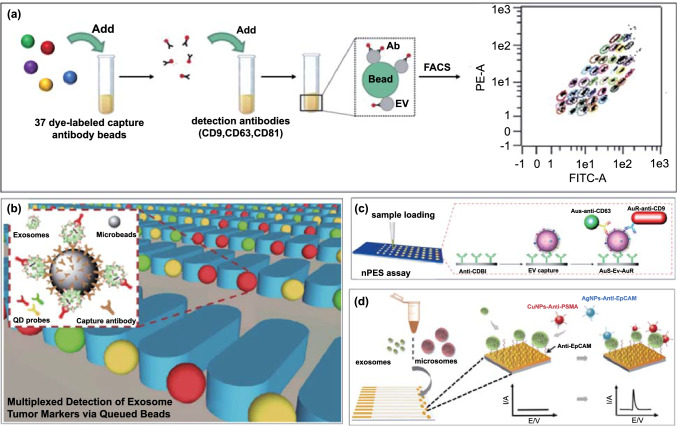
Nanoparticle coding strategy for EV multiplexing using **a** fluorophore doped beads. Reproduced with permission from Ref. [165]. Copyright 2020 AAN Publications. **b** Quantum dots. Reproduced with permission from Ref. [185]. Copyright 2019 Springer. **c** Plasmonic nanoparticles. Reproduced with permission from Ref. [177]. Copyright 2017 Springer Nature. **d** Redox active Cu and Ag nanoparticles. Reproduced with permission [179]. Copyright 2014 John Wiley & Sons

The combination of fluorescent barcoded beads with flow cytometry (FCM) is a widely applied detection approach. EVs are typically too small to be detected by FCM directly by conventional flow cytometers but an adaptation of the technique can be used to quantify multiple EV protein markers [161]. This can be generically achieved by two immune-sandwich formats, i.e. capture antibody modified microbeads-EVs-fluorescent detection antibodies [162] or capture antibody modified barcoded microbeads-EVs-allophycocyanin (APC) or phycoerythrin (PE)-conjugated detection antibodies (Fig. 10a) [163–165]. When a laser beam illuminates EVs, the scattered light is converted to an intensity-associated voltage pulse that can be quantified. Depending on marker selection, the bead EV-based FCM allows the evaluation of different subpopulations in a single sample or the comparison of EV subpopulations in different samples. Koliha et al., for example, have described a multiplex bead-based FCM platform for the analysis of different subpopulations of EVs where up to 39 EV surface proteins in different cell lines and subsets of rare cells were analysed in work demonstrating the strength of this method in profiling heterogeneous EV subpopulations [162].

A range of commercial analysis platforms are available from Becton Dickinson and Company (Cytometric Bead Array) and the partners of Luminex Corp (xMAP™), with products for in vitro diagnostics (IVD). These utilise sandwich immunoassays consisting of beads that differ in size or fluorescence intensity (colour or scattering-coded beads), conjugated with capture antibodies that are used for multiplexed immunoassays. Shi et al., for example, have employed the Luminex platform to study potential EV biomarkers in Parkinson’s disease (PD) [163]. They demonstrated that EV-associated α-synuclein is significantly higher in PD patients as compared to controls with an AUC of 0.724 (sensitivity = 76.8%, specificity = 53.5%). More recently, Vacchi et al. have used the FCM platform to explore 37 EV surface markers using the plasma-derived EV from PD, healthy controls (HC), multiple system atrophy (MSA), and Atypical Parkinsonism (AP) with tauopathy (AP-Tau) [165]. Although they used a small number of cases, this work demonstrated that the combination of CD2, CD62P and CD146 could clearly separate MSA from HC (AUC = 0.961, sensitivity = 100%, specificity = 84.2%).

It is noteworthy that aggregation of EV-bead complexes may complicate assays and lead to biased results. Nonspecific signals, additionally, remain another issue [166]. Encouragingly, new variations of flow cytometry have been developed, for example, the newly developed nano-FCM to facilitate profiling with higher resolution (~ 30 nm) and sensitivity [167, 168].

### Quantum Dots

Quantum dots (QDs) are relatively easy to synthesize with highly tunable optical properties and have accordingly been explored as optical tags for EV profiling. In a recent study, Bai et al., for example, developed a bead-based microarray for EV isolation and multiplexed tumour marker detection. EVs were isolated with anti-CD9 antibody-coated polystyrene microbeads, and tumour markers on the captured EVs were detected with antibodies decorated with quantum dot probes (i.e. QD_525 nm_, QD_585 nm_, QD_625 nm_) as shown in Fig. 10b supporting the detection of the EV proteins CEA, Cyfra21-1, and ProGRP. Using this assay, lung cancer-derived samples were observed to exhibit between 6- to 10-fold higher fluorescence intensity than endothelial cell samples, and different types of lung cancer samples showed distinct marker expression. The antibody-QD coding-based platform offers clear advantages over the conventional organic fluorophores in terms of broader excitation and more sharply defined emission signatures [169–171]. Antibody-conjugated quantum dots (i.e. QDs_605_-anti-EpCAM and QDs_655_-anti-EphA2) have also been employed with a lipophilic fluorescent dye DiO (3,3’-dihexadecyloxacarbocyanine perchlorate) reference stain to provide a quantitative readout. This introduction of normalization enabled the quantification of EV biomarkers in pancreatic cancer with higher performance. The main drawbacks of such immunostaining-based multiplexed analysis are the needs for repeated staining and the generation of signals distinct from nonspecific fluorescence.

### Plasmonic Nanoparticles

In addition to SPR, a number of localized SPR (LSPR) platforms have been applied to EVs [108, 172–174]. LSPR results from the confinement of a strong electromagnetic field exhibited by a noble metal in its nanostructure form [175, 176] and is highly sensitive to the local refractive index variation adjacent to the metallic surface. For example, Liang et al. have reported a sensitive and specific method to detect pancreatic cancer EV markers by using nanoplasmon-enhanced scattering (nPES) sensor (Fig. 10c) [177]. Specifically, anti-EphA2-modified AuNPs and anti-CD9-modified AuNRs were bound to the pre-captured EVs on an anti-CD81 coated chip surface to simultaneously assess for the presence of both membrane proteins. When the two nanoparticles were in close proximity (< 10 nm), their scattering spectra shifted and could be analysed by dark field microscopy. This coding can detect EV proteins down 0.2 ng µL^−1^ from as little as 1 µL of plasma. In this work, the authors demonstrate that the nPES assay using EphA2-EVs can distinguish pancreatic cancer from pancreatitis and healthy subjects or used to stage tumour progression and detect early responses to neoadjuvant therapy. The proposed nPES platform offers an attractive means for a rapid, purification-free and ultrasensitive assessment of EVs. Major challenges here are associated with the tuning of scattering properties (e.g. shape and size). In this regard, photonic materials with tunable structural colours that have not been applied in EV profiling could offer a solution [178].

### Electroactive Nanoparticles

Electroactive nanoparticles bearing distinct redox properties have also been utilized as tags for EV multiplexing. For example, Zhou et al. reported a microfabricated microfluidic sensor chip containing eleven individual gold electrodes that enable multiple readouts (Fig. 10d) [179]. Instead of using immunomagnetic beads for pre-enrichment of EVs within or outside the fluidic channel, the gold electrodes were directly modified with thiolated anti-EpCAM aptamer to bind EVs. Two metal nanoparticle-based redox labels, anti-EpCAM aptamer-modified silver nanoparticles (AgNPs) and anti-PSMA (prostate-specific membrane antigen) aptamer-modified copper nanoparticles (CuNPs), were applied as labels. Taking advantage of their distinct oxidation potentials (AgNPs, + 350 mV *vs.* Ag/AgCl, while the CuNPs, + 600 mV *vs.* Ag/AgCl), the direct electro-oxidation of both nanoparticles generated distinct electrochemical peaks through which EV markers could be quantified. This platform exhibited a limit of detection (LOD) of 50 EVs/sensor with a low sample input (25 μL) and a fast response (≤ 10 s) and was used to show that increased levels of PSMA and EpCAM exist in EVs from prostate cancer positive serum samples. This concept has been extended to the analysis of cancer cells by electrochemical oxidation of metal nanoparticle tags, including Ag, Cu, and Pd [180]. A similar molecular redox probe based approach has also been explored in electrochemical multiplexing. For example, separately labelled redox probes (Nile blue, ferrocene, and methylene blue with redox peak at − 0.4, − 0.2, and 0.2 V in single sweep) to graphene oxides tailored with detection antibodies to function as electrochemical signal tags in the sandwich assays were used for multiple cytokine sensing (IL-6, IL-1β, and TNF-α) [181, 182]. Guo et al*.* synthesized different ruthenium complexes to achieve an immunosensor for multiplexing cancer biomarkers (CEA, AFP, and beta-human chorionic gonadotropin) [183]. This work has extended the scope of electrochemical multiplexing and is highly adaptable for the detection of EV markers [184]. Like the optical equivalent, the limitation of this approach is the number of spectrally (electrochemically) distinct labels conveniently available.

## Conclusions and Outlook

**Fig. 11:**
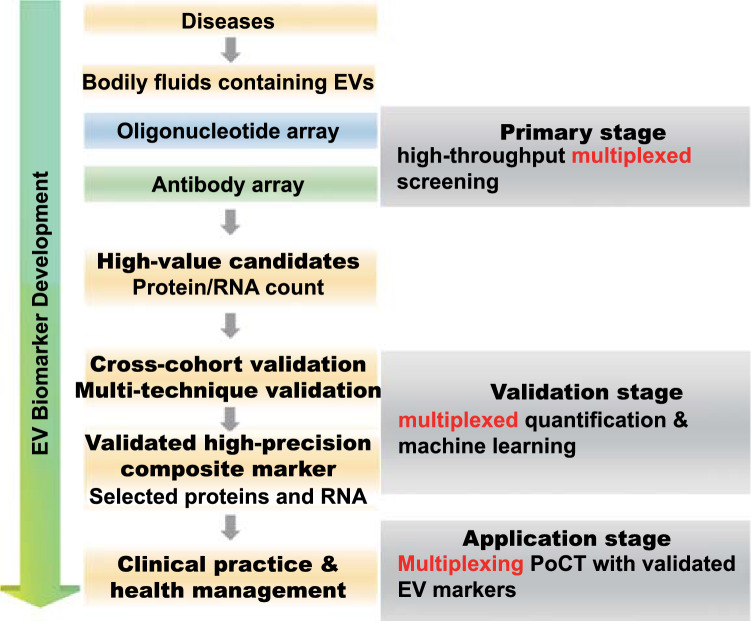
Flowchart of EV biomarker development powered by multiplexing platform

EV content such as proteins and RNA is emerging as a potentially potent source of biomarkers across diseases including cancer, cardiovascular, and neurological diseases. The development of multiplexing platforms offers a powerful toolbox to enable their clinical application. The existing strategies, i.e. chemical-, physical-, biological-, nanoparticle-based and their convergence, have been systemically reviewed and compared herein. The past decade has witnessed great progress in EV isolation and analysis, with a substantial push towards real clinical translation [186, 187], a push that is much more likely to be realized if robust multiplexed analyses can be developed and commercially scaled. Figure 11 summarises the potential use of multiplexed platforms at the different stages of EV biomarker development: these include high-throughput screening of EV biomarker candidates (*n* > 10), validation of the candidates using multiple platforms or cohorts, and low-throughput devices for the use of validated EV markers in routine clinical practice. It is notable that most methodologies highlighted here have been developed within the last few years and tested mostly in pilot studies involving only a small number of patient samples. To the best of our knowledge, no EV biomarker is yet to enter routine clinical practice. Although the field remains in its infancy, its promise is profound. Below, we discuss the main challenges in clinical translation and the potential solutions to tackle them.

### Assay Standardization

Given the diversity of detection techniques discussed in earlier sections (e.g. ECL, electrochemistry, SPR, SERS, fluorescence), assay variation and standardization across laboratories is important and remains problematic. Multiplexed platforms for the analysis of clinical samples may, additionally, suffer from variability in the quality of reagents provided from different suppliers. For example, the affinity constant (i.e. association constant) of antibodies, which may vary depending on the production method, is a key factor that influences assay sensitivity [133]. Thus, high-quality control for biologics (e.g. well-characterized antibodies from the same clone) and batch-to-batch assessment of binding parameters should be available to increase reproducibility. This also requires stringent quality control within each assay, such as the consistent detection of a generic EV marker within each sample. Such surface or internal EV markers often vary between laboratories and a broad consensus could aid the interpretation of disease-associated markers. This is especially relevant for EV biomarker validation by different laboratories using the same or different detection platforms. In addition, the quality of biosamples (e.g. blood, CSF, urine) used to extract EVs may vary between cohorts or centres due to issues at collection (e.g. haemolysis during serum extraction or blood contamination of CSF), transport and storage time-lapse or freeze–thaw cycles. Sample processing and quality control information should be rigorously reported in published studies. To date, most benchtop detection platforms are solely proof-of-concept demonstrations which rely mostly on complicated fabrication steps of the sensor and a series of optimizations performed in a well-equipped centralized facility. Hence, standardized design and fabrication protocols are needed to translate benchtop assays into bedside clinical applications [186].

### More Adaptive EV Multiplexing Strategies

#### Barcoding System

Although the concept of barcoding has been used in several EV studies (see Sect. 6.1), the high coding capacity of barcodes in labelling could revolutionise multiplexed assays and high-throughput detection. Barcode technology with facile manipulation of the barcode particle composition to endow spectroscopic, graphical, electronic, and physical codes can be employed for labelling numerous target analytes with distinguishable signal readout [187, 188]. For clinical application, barcodes serve as reporters to label multiple EV targets, thus offering a promising alternative to traditional labels for EV biomarker identification and discovery [189].

#### Chemical Nose System

Rather than using conventional bio-receptors like antibodies or aptamers, a panel of well-designed chemical ligands, e.g. cationic thiolated-AuNPs and anionic fluorescent polymer-based transducer-based complexations, could be used [190–192]. These chemical ligands contact targets generating optical signals through ligand-target interaction induced displacement, making it a promising means of evaluating EV membrane proteins especially. The latest version of such a chemical ligand system (AIE system) consists of a panel of tetraphenylethene (TPE) derivatives [193, 194] to facilitate EV profiling.

#### CRISPR/Cas System

The genome editing capacity of the CRISPR/Cas system has been investigated as a novel multiplexing platform [195–199]. Owing to the inherent programmability of Cas proteins [200], collateral activity upon multiple 20-mer barcodes can be extended to report potentially hundreds of orthogonal codes. A proof-of-concept study using CRISPR-Cas12a-mediated barcodes has been applied in the detection of urine biomarkers [197]. Massively multiplexed nucleic acid detection with Cas13-based SHERLOCK detection technology along with microfluidic chips has also been applied to both COVID-19 and other viral infections [201]. Another recent multiplexing platform combining barcoding and dCas9 has been successfully applied to multiple bacteria DNA detection [202], suggesting that these configurations have high enough flexibility and adaptability to underpin sophisticated EV analysis [200, 203, 204].

### Advanced Fabrication-Assisted High-throughput and PoC Devices

High-throughput EV profiling platforms for screening multiple analytes from numerous subjects and the low-throughput PoC devices for the detection of validated EV biomarkers are the important bottlenecks in EV biomarker development (Fig. 11). Their fabrication can, in large part, be readily solved and accelerated by advanced manufacturing [73, 205–207], e.g. photolithography-based micro-/nanofabrication, micro-electro-mechanical systems (MEMS), and 3D printing [208–211] which can integrate different functional modules (isolation, high-throughput detection) into a sample handling microfluidic devices. The most common formats of this kind have enabled the integration of EV isolation and detection as applied to membrane marker profiling, but rarely for internal EV marker measurements since an additional lysis step is needed [212, 213]. A promising alternative method to realize in situ isolation-lysis-detection could involve the incorporation of an acoustic [214, 215] or an electric field [156] module to realize the goal of “sample-in, result-out” [216, 217] within a single miniaturized device. The on-site acoustic wave lysis might be a good option to avoid the injection of a lysis buffer to the channel. This is potentially easier to operate and has been used for on-chip EV RNA analysis [215]. This “all-in-one” strategy can undoubtedly improve reproducibility by eliminating the manual handling of EV isolation before introducing them into the multiplexing device as summarized in Table 1. The integration of more sophisticated nonfouling chemistries on device interfaces should reduce false positive/negative results, especially as applied to complex samples [113, 132, 218]. The investigation of multi-dimensional markers such as RNA and proteins in one device is also of high significance. This can further reduce the handling variations across multiple detection platforms. The emerging integration of powerful algorithms could combine surface signatures and internal cargo profiles, offering more comprehensive profiling of EV markers in disease.

### High-Performance Composite Markers and Machine Learning

It is becoming clear that a combination of biomarker candidates is more likely to generate a precise readout in complex diseases since single markers have failed to achieve this objective in most studies. Composite biomarkers are expected to better reflect disease stratification or progression and provide precision diagnosis in prodromal stages. For example, we have recently demonstrated that the composite measurement of α-synuclein and clusterin in serum L1CAM-positive EVs was highly accurate (AUC = 0.98) in differentiating Parkinson’s disease from atypical parkinsonism using 735 samples from four independent cohorts. This was superior to the performance of the corresponding individual markers (~ AUC 0.82−0.86) [93]. Another report has shown that the detection of EGFR activating and T790M mutations in EV RNA and free circulating DNA offers clinically meaningful sensitivity (> 0.9) for lung cancer studies [219]. Advanced data analysis will be important in maximizing the impact of multiplexed measurements across large cohorts. Machine learning has been widely used to maximise the benefits of multi-dimensional markers, e.g. multi-omics-based biomarkers [220–222]. It offers a powerful means to handle big data especially when dealing with multiplexed profiling of individual samples from hundreds of patients. The application of advanced algorithms capable of studying the logical relationship between EV biomarkers and disease will facilitate the development of high-performance composite marker patterns.

### Multiplexing at Single-EV Level

A more detailed mapping of the surface proteins of EV subpopulations and the development of single-EV level multiplexing platforms will revolutionise the field. This powerful combination will enable an in-depth study of EV heterogeneity and, more importantly, will provide the sensitivity and resolution needed for precise monitoring of the diverse pathological processes that underpin complex diseases. Recent studies have started to investigate multiple membrane proteins or lipid content at single-EV level [51, 223]. It is worth noting that, although single-cell proteomics and sequencing have been realized, the translation of these technologies to single-EV measurements is currently challenging due to trace levels of their cargoes. The improvement of assay sensitivity will undoubtedly support breakthroughs in this area.

The development of multiplexing platforms with diverse strategies has enabled the discovery and quantitation of promising EV biomarkers over the past decade, but their translation into routinely used clinical tests is yet to be fully realized. The latter will require further standardization of current methods and higher resolution potentially at single particle level to capture EV heterogeneity and their precise association with complex diseases.

## References

[1] Colombo M, Raposo G, Théry C (2014). Biogenesis, secretion, and intercellular interactions of exosomes and other extracellular vesicles. Annu. Rev. Cell Dev. Biol..

[2] Loh YP (2021). Advances in EV isolation technology and function. Extracell. Vesicles Circ. Nucleic Acids.

[3] Fan Z, Yu J, Lin J, Liu Y, Liao Y (2019). Exosome-specific tumor diagnosis via biomedical analysis of exosome-containing microrna biomarkers. Analyst.

[4] Ragni E, Orfei CP, Papait A, Girolamo L (2021). Comparison of miRNA cargo in human adipose-tissue Vs amniotic-membrane derived mesenchymal stromal cells extracellular vesicles for osteoarthritis treatment. Extracell. Vesicles Circ. Nucleic Acids.

[5] Xiong H, Huang Z, Yang Z, Lin Q, Yang B (2021). Recent progress in detection and profiling of cancer cell-derived exosomes. Small.

[6] Cui S, Cheng Z, Qin W, Jiang L (2018). Exosomes as a liquid biopsy for lung cancer. Lung Cancer.

[7] Hu T, Wolfram J, Srivastava S (2021). Extracellular vesicles in cancer detection: hopes and hypes. Trends in Cancer.

[8] Fan Z, Xiao K, Lin J, Liao Y, Huang X (2019). Functionalized DNA enables programming exosomes/vesicles for tumor imaging and therapy. Small.

[9] Tamura T, Yoshioka Y, Sakamoto S, Ichikawa T, Ochiya T (2021). Extracellular vesicles as a promising biomarker resource in liquid biopsy for cancer. Extracell. Vesicles Circ. Nucleic Acids.

[10] Zhang Y, Ding N, Xie S, Ding Y, Huang M (2021). Identification of important extracellular vesicle RNA molecules related to sperm motility and prostate cancer. Extracell. Vesicles Circ. Nucleic Acids.

[11] Tomlinson PR, Zheng Y, Fischer R, Heidasch R, Gardiner C (2015). Identification of distinct circulating exosomes in Parkinson's disease. Ann. Clin. Transl. Neurol..

[12] Li Q, Tofaris GK, Davis JJ (2017). Concentration-normalized electroanalytical assaying of exosomal markers. Anal. Chem..

[13] Tofaris GK (2017). A critical assessment of exosomes in the pathogenesis and stratification of Parkinson’s disease. J. Parkinsons. Dis..

[14] Xiao L, Hareendran S, Loh YP (2021). Function of exosomes in neurological disorders and brain tumors. Extracell. Vesicles Circ. Nucleic Acids.

[15] Jiang C, Hopfner F, Berg D, Hu MT, Pilotto A (2021). Validation of α-synuclein in L1CAM-immunocaptured exosomes as a biomarker for the stratification of parkinsonian syndromes. Mov. Disord..

[16] Bei Y, Das S, Rodosthenous RS, Holvoet P, Vanhaverbeke M (2017). Extracellular vesicles in cardiovascular theranostics. Theranostics.

[17] Jansen F, Nickenig G, Werner N (2017). Extracellular vesicles in cardiovascular disease. Circul. Res..

[18] Shao H, Im H, Castro CM, Breakefield X, Weissleder R (2018). New technologies for analysis of extracellular vesicles. Chem. Rev..

[19] Xu H, Ye BC (2020). Advances in biosensing technologies for analysis of cancer-derived exosomes. TrAC Trends Anal. Chem..

[20] Zhou S, Yang Y, Wu Y, Liu S (2021). Review: multiplexed profiling of biomarkers in extracellular vesicles for cancer diagnosis and therapy monitoring. Anal. Chim. Acta.

[21] Nakamura A, Kaneko N, Villemagne VL, Kato T, Doecke J (2018). High performance plasma amyloid-Β biomarkers for Alzheimer’s disease. Nature.

[22] Jin D, Peng XX, Qin Y, Wu P, Lu H (2020). Multivalence-actuated DNA nanomachines enable bicolor exosomal phenotyping and PD-L1-guided therapy monitoring. Anal. Chem..

[23] Chen Q, Sun T, Jiang C (2021). Recent advancements in nanomedicine for ‘cold’ tumor immunotherapy. Nano-Micro Lett..

[24] Zhang M, Jin K, Gao L, Zhang Z, Li F (2018). Methods and technologies for exosome isolation and characterization. Small Methods.

[25] Ziaei P, Berkman CE, Norton MG (2018). Isolation and detection of tumor-derived extracellular vesicles. ACS Appl. Nano Mater..

[26] Zhao Z, Wijerathne H, Godwin AK, Soper SA (2021). Isolation and analysis methods of extracellular vesicles (EVs). Extracell. Vesicles Circ. Nucleic Acids.

[27] Abramowicz A, Widlak P, Pietrowska M (2016). Proteomic analysis of exosomal cargo: the challenge of high purity vesicle isolation. Mol. Biosyst..

[28] Choi DS, Kim DK, Kim YK, Gho YS (2015). Proteomics of extracellular vesicles: exosomes and ectosomes. Mass Spectrom. Rev..

[29] Wu S, Li Y, Ding W, Xu L, Ma Y (2020). Recent advances of persistent luminescence nanoparticles in bioapplications. Nano-Micro Lett..

[30] Zong C, Xu M, Xu LJ, Wei T, Ma X (2018). Surface-enhanced Raman spectroscopy for bioanalysis: reliability and challenges. Chem. Rev..

[31] Plou J, García I, Charconnet M, Astobiza I, García-Astrain C (2020). Multiplex SERS detection of metabolic alterations in tumor extracellular media. Adv. Funct. Mater..

[32] Zheng Y, Jiang C, Ng SH, Lu Y, Han F (2016). Unclonable plasmonic security labels achieved by shadow-mask-lithography-assisted self-assembly. Adv. Mater..

[33] Ji Z, Zhang C, Ye Y, Ji J, Dong H (2021). Magnetically enhanced liquid SERS for ultrasensitive analysis of bacterial and SARS-Cov-2 biomarkers. Front. Bioeng. Biotechnol..

[34] Wang Y, Zeng S, Crunteanu A, Xie Z, Humbert G (2021). Targeted sub-attomole cancer biomarker detection based on phase singularity 2D nanomaterial-enhanced plasmonic biosensor. Nano-Micro Lett..

[35] Shin H, Jeong H, Park J, Hong S, Choi Y (2018). Correlation between cancerous exosomes and protein markers based on surface-enhanced Raman spectroscopy (SERS) and principal component analysis (PCA). ACS Sens..

[36] Zhang W, Jiang L, Diefenbach RJ, Campbell DH, Walsh BJ (2020). Enabling sensitive phenotypic profiling of cancer-derived small extracellular vesicles using surface-enhanced Raman spectroscopy nanotags. ACS Sens..

[37] Wang Z, Zong S, Wang Y, Li N, Li L (2018). Screening and multiple detection of cancerous exosomes using a SERS-based method. Nanoscale.

[38] Ning CF, Wang L, Tian YF, Yin BC, Ye BC (2020). Multiple and sensitive SERS detection of cancer-related exosomes based on gold–silver bimetallic nanotrepangs. Analyst.

[39] Wang J, Wuethrich A, Sina AAI, Lane RE, Lin LL (2020). Tracking extracellular vesicle phenotypic changes enables treatment monitoring in melanoma. Sci. Adv..

[40] Huang G, Lin G, Zhu Y, Duan W, Jin D (2020). Emerging technologies for profiling extracellular vesicle heterogeneity. Lab Chip.

[41] Cialla D, März A, Böhme R, Theil F, Weber K (2012). Surface-enhanced Raman spectroscopy (SERS): progress and trends. Anal. Bioanal. Chem..

[42] Cialla-May D, Zheng XS, Weber K, Popp J (2017). Recent progress in surface-enhanced Raman spectroscopy for biological and biomedical applications: from cells to clinics. Chem. Soc. Rev..

[43] Zhao Z, Yang Y, Zeng Y, He M (2016). A microfluidic Exosearch chip for multiplexed exosome detection towards blood-based ovarian cancer diagnosis. Lab Chip.

[44] Deng M, Jiang C, Jia L (2013). N-Methylimidazolium modified magnetic particles as adsorbents for solid phase extraction of genomic deoxyribonucleic acid from genetically modified soybeans. Anal. Chim. Acta.

[45] Guan Y, Jiang C, Hu C, Jia L (2010). Preparation of multi-walled carbon nanotubes functionalized magnetic particles by sol-gel technology and its application in extraction of estrogens. Talanta.

[46] Lin S, Yu Z, Chen D, Wang Z, Miao J (2020). Progress in microfluidics-based exosome separation and detection technologies for diagnostic applications. Small.

[47] Xu S, Jiang C, Lin Y, Jia L (2012). Magnetic nanoparticles modified with polydimethylsiloxane and multi-walled carbon nanotubes for solid-phase extraction of fluoroquinolones. Microchim. Acta.

[48] Fang S, Tian H, Li X, Jin D, Li X (2017). Clinical application of a microfluidic chip for immunocapture and quantification of circulating exosomes to assist breast cancer diagnosis and molecular classification. PLoS ONE.

[49] He M, Crow J, Roth M, Zeng Y, Godwin AK (2014). Integrated immunoisolation and protein analysis of circulating exosomes using microfluidic technology. Lab Chip.

[50] Wang S, Khan A, Huang R, Ye S, Di K (2020). Recent advances in single extracellular vesicle detection methods. Biosens. Bioelectron..

[51] Lee K, Fraser K, Ghaddar B, Yang K, Kim E (2018). Multiplexed profiling of single extracellular vesicles. ACS Nano.

[52] Cavallaro S, Pevere F, Stridfeldt F, Görgens A, Paba C (2021). Multiparametric profiling of single nanoscale extracellular vesicles by combined atomic force and fluorescence microscopy: correlation and heterogeneity in their molecular and biophysical features. Small.

[53] Wang H, Chen H, Huang Z, Li T, Deng A (2018). DAase I enzyme-aided fluorescence signal amplification based on graphene oxide-DNA aptamer interactions for colorectal cancer exosome detection. Talanta.

[54] Li B, Liu C, Pan W, Shen J, Guo J (2020). Facile fluorescent aptasensor using aggregation-induced emission luminogens for exosomal proteins profiling towards liquid biopsy. Biosens. Bioelectron..

[55] Xia Y, Liu M, Wang L, Yan A, He W (2017). A visible and colorimetric aptasensor based on DNA-capped single-walled carbon nanotubes for detection of exosomes. Biosens. Bioelectron..

[56] Jin D, Yang F, Zhang Y, Liu L, Zhou Y (2018). ExoAPP: exosome-oriented, aptamer nanoprobe-enabled surface proteins profiling and detection. Anal. Chem..

[57] Liu C, Zhao J, Tian F, Cai L, Zhang W (2019). Low-cost thermophoretic profiling of extracellular-vesicle surface proteins for the early detection and classification of cancers. Nat. Biomed. Eng..

[58] Tian F, Han Z, Deng J, Liu C, Sun J (2021). Thermomicrofluidics for biosensing applications. VIEW.

[59] Lin B, Tian T, Lu Y, Liu D, Huang M (2021). Tracing tumor-derived exosomal PD-L1 by dual-aptamer activated proximity-induced droplet digital PCR. Angew. Chem. Int. Ed..

[60] Wu D, Yan J, Shen X, Sun Y, Thulin M (2019). Profiling surface proteins on individual exosomes using a proximity barcoding assay. Nat. Comm..

[61] Löf L, Ebai T, Dubois L, Wik L, Ronquist KG (2016). Detecting individual extracellular vesicles using a multicolor in situ proximity ligation assay with flow cytometric readout. Sci. Rep..

[62] Zhang J, Shi J, Zhang H, Zhu Y, Liu W (2020). Localized fluorescent imaging of multiple proteins on individual extracellular vesicles using rolling circle amplification for cancer diagnosis. J. Extracell. Vesicles.

[63] Wu X, Zhao H, Natalia A, Lim CZJ, Ho NRY (2020). Exosome-templated nanoplasmonics for multiparametric molecular profiling. Sci. Adv..

[64] Yeh EC, Fu CC, Hu L, Thakur R, Feng J (2017). Self-powered integrated microfluidic point-of-care low-cost enabling (SIMPLE) chip. Sci. Adv..

[65] Yelleswarapu V, Buser JR, Haber M, Baron J, Inapuri E (2019). Mobile platform for rapid sub–picogram-per-milliliter, multiplexed, digital droplet detection of proteins. PNAS.

[66] Shu B, Lin L, Wu B, Huang E, Wang Y (2021). A pocket-sized device automates multiplexed point-of-care RNA testing for rapid screening of infectious pathogens. Biosens. Bioelectron..

[67] Tian T, Shu B, Jiang Y, Ye M, Liu L (2021). An ultralocalized cas13a assay enables universal and nucleic acid amplification-free single-molecule RNA diagnostics. ACS Nano.

[68] Lim CZJ, Zhang Y, Chen Y, Zhao H, Stephenson MC (2019). Subtyping of circulating exosome-bound amyloid β reflects brain plaque deposition. Nat. Comm..

[69] Li Y, Deng J, Han Z, Liu C, Tian F (2021). Molecular identification of tumor-derived extracellular vesicles using thermophoresis-mediated DNA computation. J. Am. Chem. Soc..

[70] Zhao J, Liu C, Li Y, Ma Y, Deng J (2020). Thermophoretic detection of exosomal microRNAs by nanoflares. J. Am. Chem. Soc..

[71] Schwarzenbach H, Nishida N, Calin GA, Pantel K (2014). Clinical relevance of circulating cell-free microRNAs in cancer. Nat. Rev. Clin. Oncol..

[72] Chevillet JR, Kang Q, Ruf IK, Briggs HA, Vojtech LN (2014). Quantitative and stoichiometric analysis of the microRNA content of exosomes. PNAS.

[73] Liu G, Jiang C, Lin X, Yang Y (2021). Point-of-care detection of cytokines in cytokine storm management and beyond: significance and challenges. VIEW.

[74] Lee JH, Kim JA, Jeong S, Rhee WJ (2016). Simultaneous and multiplexed detection of exosome micrornas using molecular beacons. Biosens. Bioelectron..

[75] Lee J, Kwon MH, Kim JA, Rhee WJ (2018). Detection of exosome miRNAs using molecular beacons for diagnosing prostate cancer. Artif. Cells Nanomed. Biotechnol..

[76] Yang HC, Rhee WJ (2021). Single step in situ detection of surface protein and microRNA in clustered extracellular vesicles using flow cytometry. J. Clin. Med..

[77] Zhou S, Hu T, Han G, Wu Y, Hua X (2020). Accurate cancer diagnosis and stage monitoring enabled by comprehensive profiling of different types of exosomal biomarkers: surface proteins and miRNAs. Small.

[78] Liu C, Kannisto E, Yu G, Yang Y, Reid ME (2020). Non-invasive detection of exosomal microRNAs via tethered cationic lipoplex nanoparticles (tCLN) biochip for lung cancer early detection. Front. Genet..

[79] Yang Y, Kannisto E, Yu G, Reid ME, Patnaik SK (2018). An immuno-biochip selectively captures tumor-derived exosomes and detects exosomal RNAs for cancer diagnosis. ACS Appl. Mater. Interfaces.

[80] Zhou J, Wu Z, Hu J, Yang D, Chen X (2020). High-throughput single-EV liquid biopsy: rapid, simultaneous, and multiplexed detection of nucleic acids, proteins, and their combinations. Sci. Adv..

[81] Wu Y, Kwak KJ, Agarwal K, Marras A, Wang C (2013). Detection of extracellular RNAs in cancer and viral infection via tethered cationic lipoplex nanoparticles containing molecular beacons. Anal. Chem..

[82] Wang X, Kwak KJ, Yang Z, Zhang A, Zhang X (2018). Extracellular mRNA detected by molecular beacons in tethered lipoplex nanoparticles for diagnosis of human hepatocellular carcinoma. PLoS ONE.

[83] Ghassemi P, Wang B, Wang J, Wang Q, Chen Y (2017). Evaluation of mobile phone performance for near-infrared fluorescence imaging. IEEE Trans. Biomed. Eng..

[84] Vaidyanathan R, Naghibosadat M, Rauf S, Korbie D, Carrascosa LG (2014). Detecting exosomes specifically: a multiplexed device based on alternating current electrohydrodynamic induced nanoshearing. Anal. Chem..

[85] Liu C, Zhao J, Tian F, Chang J, Zhang W (2019). λ-DNA- and aptamer-mediated sorting and analysis of extracellular vesicles. J. Am. Chem. Soc..

[86] Im H, Shao H, Park YI, Peterson VM, Castro CM (2014). Label-free detection and molecular profiling of exosomes with a nano-plasmonic sensor. Nat. Biotechnol..

[87] Liu W, Li J, Wu Y, Xing S, Lai Y (2018). Target-induced proximity ligation triggers recombinase polymerase amplification and transcription-mediated amplification to detect tumor-derived exosomes in nasopharyngeal carcinoma with high sensitivity. Biosens. Bioelectron..

[88] Jiang Y, Shi M, Liu Y, Wan S, Cui C (2017). Aptamer/AuNP biosensor for colorimetric profiling of exosomal proteins. Angew. Chem. Int. Ed..

[89] Lyu Y, Cui D, Huang J, Fan W, Miao Y (2019). Near-infrared afterglow semiconducting nano-polycomplexes for the multiplex differentiation of cancer exosomes. Angew. Chem. Int. Ed..

[90] Chiu YJ, Cai W, Shih YRV, Lian I, Lo YH (2016). A single-cell assay for time lapse studies of exosome secretion and cell behaviors. Small.

[91] Mori K, Hirase M, Morishige T, Takano E, Sunayama H (2019). A pretreatment-free, polymer-based platform prepared by molecular imprinting and post-imprinting modifications for sensing intact exosomes. Angew. Chem. Int. Ed..

[92] Jeong S, Park J, Pathania D, Castro CM, Weissleder R (2016). Integrated magneto–electrochemical sensor for exosome analysis. ACS Nano.

[93] Jiang C, Hopfner F, Katsikoudi A, Hein R, Catli C (2020). Serum neuronal exosomes predict and differentiate Parkinson’s disease from atypical parkinsonism. J. Neurol. Neurosurg. Psychiatry.

[94] Park J, Im H, Hong S, Castro CM, Weissleder R (2018). Analyses of intravesicular exosomal proteins using a nano-plasmonic system. ACS Photonics.

[95] Kwizera EA, O'Connor R, Vinduska V, Williams M, Butch ER (2018). Molecular detection and analysis of exosomes using surface-enhanced Raman scattering gold nanorods and a miniaturized device. Theranostics.

[96] Zhu L, Wang K, Cui J, Liu H, Bu X (2014). Label-free quantitative detection of tumor-derived exosomes through surface plasmon resonance imaging. Anal. Chem..

[97] Rodrigues M, Richards N, Ning B, Lyon CJ, Hu TY (2019). Rapid lipid-based approach for normalization of quantum-dot-detected biomarker expression on extracellular vesicles in complex biological samples. Nano Lett..

[98] Takeuchi T, Mori K, Sunayama H, Takano E, Kitayama Y (2020). Antibody-conjugated signaling nanocavities fabricated by dynamic molding for detecting cancers using small extracellular vesicle markers from tears. J. Am. Chem. Soc..

[99] Zhang P, Zhou X, Zeng Y (2019). Multiplexed immunophenotyping of circulating exosomes on nano-engineered exoprofile chip towards early diagnosis of cancer. Chem. Sci..

[100] Zhou S, Hu T, Zhang F, Tang D, Li D (2020). Integrated microfluidic device for accurate extracellular vesicle quantification and protein markers analysis directly from human whole blood. Anal. Chem..

[101] An Y, Li R, Zhang F, He P (2020). Magneto-mediated electrochemical sensor for simultaneous analysis of breast cancer exosomal proteins. Anal. Chem..

[102] Yang KS, Im H, Hong S, Pergolini I, Castillo AF (2017). Multiparametric plasma EV profiling facilitates diagnosis of pancreatic malignancy. Sci. Transl. Med..

[103] Cheng HL, Fu CY, Kuo WC, Chen YW, Chen YS (2018). Detecting mirna biomarkers from extracellular vesicles for cardiovascular disease with a microfluidic system. Lab Chip.

[104] Wang H, He D, Wan K, Sheng X, Cheng H (2020). In situ multiplex detection of serum exosomal micrornas using all-in-one biosensor for breast cancer diagnosis. Analyst.

[105] Jang M, Choi G, Choi YY, Lee JE, Jung JH (2019). Extracellular vesicle (EV)-polyphenol nanoaggregates for microrna-based cancer diagnosis. NPG Asia Mater..

[106] Wang Z, Sun X, Natalia A, Tang CSL, Ang CBT (2020). Dual-selective magnetic analysis of extracellular vesicle glycans. Matter.

[107] Rupert DLM, Lässer C, Eldh M, Block S, Zhdanov VP (2014). Determination of exosome concentration in solution using surface plasmon resonance spectroscopy. Anal. Chem..

[108] Thakur A, Qiu G, Ng SP, Guan J, Yue J (2017). Direct detection of two different tumor-derived extracellular vesicles by SAM-AuNIs LSPR biosensor. Biosens. Bioelectron..

[109] Gool EL, Stojanovic I, Schasfoort RBM, Sturk A, Leeuwen TG (2017). Surface plasmon resonance is an analytically sensitive method for antigen profiling of extracellular vesicles. Clin. Chem..

[110] Khan MA, Zhu Y, Yao Y, Zhang P, Agrawal A (2020). Impact of metal crystallinity-related morphologies on the sensing performance of plasmonic nanohole arrays. Nanoscale.

[111] Liu C, Yang Y, Wu Y (2018). Recent advances in exosomal protein detection via liquid biopsy biosensors for cancer screening, diagnosis, and prognosis. AAPS J..

[112] Taufik S, Barfidokht A, Alam MT, Jiang C, Parker SG (2016). An antifouling electrode based on electrode–organic layer–nanoparticle constructs: electrodeposited organic layers versus self-assembled monolayers. J. Electroanal. Chem..

[113] Jiang C, Wang G, Hein R, Liu N, Luo X (2020). Antifouling strategies for selective in vitro and in vivo sensing. Chem. Rev..

[114] Wu W, Yu X, Wu J, Wu T, Fan Y (2021). Surface plasmon resonance imaging-based biosensor for multiplex and ultrasensitive detection of NSCLC-associated exosomal miRNAs using DNA programmed heterostructure of Au-on-Ag. Biosens. Bioelectron..

[115] Huang JA, Mousavi MZ, Zhao Y, Hubarevich A, Omeis F (2019). SERS discrimination of single DNA bases in single oligonucleotides by electro-plasmonic trapping. Nat. Commun..

[116] Huang JA, Mousavi MZ, Giovannini G, Zhao Y, Hubarevich A (2020). Multiplexed discrimination of single amino acid residues in polypeptides in a single SERS hot spot. Angew. Chem. Int. Ed..

[117] Pan S, Zhang Y, Natalia A, Lim CZJ, Ho NRY (2021). Extracellular vesicle drug occupancy enables real-time monitoring of targeted cancer therapy. Nat. Nanotechnol..

[118] Min J, Son T, Hong JS, Cheah PS, Wegemann A (2020). Plasmon-enhanced biosensing for multiplexed profiling of extracellular vesicles. Adv. Biosyst..

[119] Su Q, Jiang C, Gou D, Long Y (2021). Surface plasmon-assisted fluorescence enhancing and quenching: from theory to application. ACS Appl. Bio Mater..

[120] Chin LK, Son T, Hong JS, Liu AQ, Skog J (2020). Plasmonic sensors for extracellular vesicle analysis: from scientific development to translational research. ACS Nano.

[121] Aoki H, Corn RM, Matthews B (2019). Microrna detection on microsensor arrays by SPR imaging measurements with enzymatic signal enhancement. Biosens. Bioelectron..

[122] Zhou C, Zou H, Sun C, Ren D, Chen J (2018). Signal amplification strategies for DNA-based surface plasmon resonance biosensors. Biosensors Bioelectron..

[123] Jørgensen M, Bæk R, Pedersen S, Søndergaard EK, Kristensen SR (2013). Extracellular vesicle (EV) array: microarray capturing of exosomes and other extracellular vesicles for multiplexed phenotyping. J. Extracell. Vesicles.

[124] Zaborowski MP, Lee K, Na YJ, Sammarco A, Zhang X (2019). Methods for systematic identification of membrane proteins for specific capture of cancer-derived extracellular vesicles. Cell Rep..

[125] Jørgensen MM, Bæk R, Varming K (2015). Potentials and capabilities of the extracellular vesicle (EV) array. J. Extracell. Vesicles.

[126] Xu L, Shoaei N, Jahanpeyma F, Zhao J, Azimzadeh M (2020). Optical, electrochemical and electrical (Nano) biosensors for detection of exosomes: a comprehensive overview. Biosens. Bioelectron..

[127] Xu H, Liao C, Zuo P, Liu Z, Ye BC (2018). Magnetic-based microfluidic device for on-chip isolation and detection of tumor-derived exosomes. Anal. Chem..

[128] Tian F, Liu C, Lin L, Chen Q, Sun J (2019). Microfluidic analysis of circulating tumor cells and tumor-derived extracellular vesicles. TrAC Trends Anal. Chem..

[129] Park J, Lin HY, Assaker JP, Jeong S, Huang CH (2017). Integrated kidney exosome analysis for the detection of kidney transplant rejection. ACS Nano.

[130] Tang CK, Vaze A, Shen M, Rusling JF (2016). High-throughput electrochemical microfluidic immunoarray for multiplexed detection of cancer biomarker proteins. ACS Sens..

[131] Goda T, Masuno K, Nishida J, Kosaka N, Ochiya T (2012). A label-free electrical detection of exosomal micrornas using microelectrode array. Chem. Comm..

[132] Jiang C, Alam MT, Silva SM, Taufik S, Fan S (2016). Unique sensing interface that allows the development of an electrochemical immunosensor for the detection of tumor necrosis factor α in whole blood. ACS Sens..

[133] Fu Y, Jiang C, Tofaris GK, Davis JJ (2020). Facile impedimetric analysis of neuronal exosome markers in Parkinson’s disease diagnostics. Anal. Chem..

[134] Jiang C, Silva SM, Fan S, Wu Y, Alam MT (2017). Aryldiazonium salt derived mixed organic layers: from surface chemistry to their applications. J. Electroanal. Chem..

[135] C. Jiang, *Protein-Resistant Electrode for Biosensing*. PhD Thesis (2016).

[136] Jiang C, Alam MT, Parker SG, Gooding JJ (2015). Zwitterionic phenyl phosphorylcholine on indium tin oxide: a low-impedance protein-resistant platform for biosensing. Electroanalysis.

[137] Patil SB, Al-Jehani RM, Etayash H, Turbe V, Jiang K (2018). Modified cantilever arrays improve sensitivity and reproducibility of nanomechanical sensing in living cells. Commun. Biol..

[138] Arlett JL, Myers EB, Roukes ML (2011). Comparative advantages of mechanical biosensors. Nat. Nanotechnol..

[139] Olcum S, Cermak N, Wasserman SC, Christine KS, Atsumi H (2014). Weighing nanoparticles in solution at the attogram scale. PNAS.

[140] Etayash H, McGee A, Kaur K, Thundat T (2016). Nanomechanical sandwich assay for multiple cancer biomarkers in breast cancer cell-derived exosomes. Nanoscale.

[141] Daaboul GG, Gagni P, Benussi L, Bettotti P, Ciani M (2016). Digital detection of exosomes by interferometric imaging. Sci. Rep..

[142] Oliveira-Rodríguez M, Serrano-Pertierra E, García AC, López-Martín S, Yañez-Mo M (2017). Point-of-care detection of extracellular vesicles: sensitivity optimization and multiple-target detection. Biosens. Bioelectron..

[143] Daaboul GG, Lopez CA, Chinnala J, Goldberg BB, Connor JH (2014). Digital sensing and sizing of vesicular stomatitis virus pseudotypes in complex media: a model for Ebola and Marburg detection. ACS Nano.

[144] Gebauer P, Malá Z, Boček P (2009). Recent progress in analytical capillary ITP. Electrophoresis.

[145] Guo S, Xu J, Estell AP, Ivory CF, Du D (2020). Paper-based ITP technology: an application to specific cancer-derived exosome detection and analysis. Biosens. Bioelectron..

[146] Shirshahi V, Liu G (2021). Enhancing the analytical performance of paper lateral flow assays: from chemistry to engineering. TrAC Trends Anal. Chem..

[147] Baharfar M, Rahbar M, Tajik M, Liu G (2020). Engineering strategies for enhancing the performance of electrochemical paper-based analytical devices. Biosens. Bioelectron..

[148] Liu L, Yang D, Liu G (2019). Signal amplification strategies for paper-based analytical devices. Biosens. Bioelectron..

[149] Luo Z, Lv T, Zhu K, Li Y, Wang L (2020). Paper-based ratiometric fluorescence analytical devices towards point-of-care testing of human serum albumin. Angew. Chem. Int. Ed..

[150] Jiang C, Alam MT, Parker SG, Darwish N, Gooding JJ (2016). Strategies to achieve control over the surface ratio of two different components on modified electrodes using aryldiazonium salts. Langmuir.

[151] Zhang X, Li Q, Jin X, Jiang C, Lu Y (2015). Quantitative determination of target gene with electrical sensor. Sci. Rep..

[152] Zhang S, Jiang C, Jia L (2011). Tetrabutylammonium phosphate-assisted separation of multiplex polymerase chain reaction products in non-gel sieving capillary electrophoresis. Anal. Biochem..

[153] Jiang C, Xu S, Zhang S, Jia L (2012). Chitosan functionalized magnetic particle-assisted detection of genetically modified soybeans based on polymerase chain reaction and capillary electrophoresis. Anal. Biochem..

[154] Song C, Chen W, Kuang J, Yao Y, Tang S (2021). Recent advances in the detection of multiple micrornas. TrAC Trends Anal. Chem..

[155] Singh N, Huang L, Wang DB, Shao N, Zhang XE (2020). Simultaneous detection of a cluster of differentiation markers on leukemia-derived exosomes by multiplex immuno-polymerase chain reaction via capillary electrophoresis analysis. Anal. Chem..

[156] Jha SK, Chand R, Han D, Jang YC, Ra GS (2012). An integrated PCR microfluidic chip incorporating aseptic electrochemical cell lysis and capillary electrophoresis amperometric DNA detection for rapid and quantitative genetic analysis. Lab Chip.

[157] Ko J, Wang Y, Carlson JCT, Marquard A, Gungabeesoon J (2020). Single extracellular vesicle protein analysis using immuno-droplet digital polymerase chain reaction amplification. Adv. Biosyst..

[158] Tian Q, He C, Liu G, Zhao Y, Hui L (2018). Nanoparticle counting by microscopic digital detection: selective quantitative analysis of exosomes via surface-anchored nucleic acid amplification. Anal. Chem..

[159] Ko J, Wang Y, Sheng K, Weitz DA, Weissleder R (2021). Sequencing-based protein analysis of single extracellular vesicles. ACS Nano.

[160] Gaňová M, Zhang H, Zhu H, Korabečná M, Neužil P (2021). Multiplexed digital polymerase chain reaction as a powerful diagnostic tool. Biosens. Bioelectron..

[161] Jara-Acevedo R, Campos-Silva C, Valés-Gómez M, Yáñez-Mó M, Suárez H (2019). Exosome beads array for multiplexed phenotyping in cancer. J. Proteomics.

[162] Koliha N, Wiencek Y, Heider U, Jüngst C, Kladt N (2016). A novel multiplex bead-based platform highlights the diversity of extracellular vesicles. J. Extracell. Vesicles.

[163] Shi M, Liu C, Cook TJ, Bullock KM, Zhao Y (2014). Plasma exosomal α-synuclein is likely CNS-derived and increased in Parkinson’s disease. Acta Neuropathol..

[164] Wiklander OPB, Bostancioglu RB, Welsh JA, Zickler AM, Murke F (2018). Systematic methodological evaluation of a multiplex bead-based flow cytometry assay for detection of extracellular vesicle surface signatures. Front. Immunol..

[165] Vacchi E, Burrello J, Di Silvestre D, Burrello A, Bolis S (2020). Immune profiling of plasma-derived extracellular vesicles identifies parkinson disease. Neurol. Neuroimmunol..

[166] Marcoux G, Duchez AC, Cloutier N, Provost P, Nigrovic PA (2016). Revealing the diversity of extracellular vesicles using high-dimensional flow cytometry analyses. Sci. Rep..

[167] Ma L, Zhu S, Tian Y, Zhang W, Wang S (2016). Label-free analysis of single viruses with a resolution comparable to that of electron microscopy and the throughput of flow cytometry. Angew. Chem. Int. Ed..

[168] Tian Y, Ma L, Gong M, Su G, Zhu S (2018). Protein profiling and sizing of extracellular vesicles from colorectal cancer patients via flow cytometry. ACS Nano.

[169] Chan WCW, Maxwell DJ, Gao X, Bailey RE, Han M (2002). Luminescent quantum dots for multiplexed biological detection and imaging. Curr. Opin. Biotechnol..

[170] Cheng X, Lowe SB, Reece PJ, Gooding JJ (2014). Colloidal silicon quantum dots: from preparation to the modification of self-assembled monolayers (SAMs) for bio-applications. Chem. Soc. Rev..

[171] Cheng X, Hinde E, Owen DM, Lowe SB, Reece PJ (2015). Enhancing quantum dots for bioimaging using advanced surface chemistry and advanced optical microscopy: application to silicon quantum dots (SiQDs). Adv. Mater..

[172] Taton TA, Lu G, Mirkin CA (2001). Two-color labeling of oligonucleotide arrays via size-selective scattering of nanoparticle probes. J. Am. Chem. Soc..

[173] Noto GD, Bugatti A, Zendrini A, Mazzoldi EL, Montanelli A (2016). Merging colloidal nanoplasmonics and surface plasmon resonance spectroscopy for enhanced profiling of multiple myeloma-derived exosomes. Biosens. Bioelectron..

[174] Rica R, Stevens MM (2012). Plasmonic elisa for the ultrasensitive detection of disease biomarkers with the naked eye. Nat. Nanotechnol..

[175] Zheng Y, Soeriyadi AH, Rosa L, Ng SH, Bach U (2015). Reversible gating of smart plasmonic molecular traps using thermoresponsive polymers for single-molecule detection. Nat. Commun..

[176] Zheng Y, Thai T, Reineck P, Qiu L, Guo Y (2013). DNA-directed self-assembly of core-satellite plasmonic nanostructures: a highly sensitive and reproducible near-IR SERS sensor. Adv. Funct. Mater..

[177] Liang K, Liu F, Fan J, Sun D, Liu C (2017). Nanoplasmonic quantification of tumour-derived extracellular vesicles in plasma microsamples for diagnosis and treatment monitoring. Nat. Biomed. Eng..

[178] Zhao Y, Xie Z, Gu H, Zhu C, Gu Z (2012). Bio-inspired variable structural color materials. Chem. Soc. Rev..

[179] Zhou YG, Mohamadi RM, Poudineh M, Kermanshah L, Ahmed S (2016). Interrogating circulating microsomes and exosomes using metal nanoparticles. Small.

[180] Wan Y, Zhou YG, Poudineh M, Safaei TS, Mohamadi RM (2014). Highly specific electrochemical analysis of cancer cells using multi-nanoparticle labeling. Angew. Chem. Int. Ed..

[181] Wei H, Ni S, Cao C, Yang G, Liu G (2018). Graphene oxide signal reporter based multifunctional immunosensing platform for amperometric profiling of multiple cytokines in serum. ACS Sens..

[182] Shen Z, Huang J, Wei H, Niu H, Li B (2020). Validation of an in vivo electrochemical immunosensing platform for simultaneous detection of multiple cytokines in Parkinson’s disease mice model. Bioelectrochemistry.

[183] Guo W, Ding H, Gu C, Liu Y, Jiang X (2018). Potential-resolved multicolor electrochemiluminescence for multiplex immunoassay in a single sample. J. Am. Chem. Soc..

[184] Lv W, Ye H, Yuan Z, Liu X, Chen X (2020). Recent advances in electrochemiluminescence-based simultaneous detection of multiple targets. TrAC Trends Anal. Chem..

[185] Bai Y, Lu Y, Wang K, Cheng Z, Qu Y (2019). Rapid isolation and multiplexed detection of exosome tumor markers via queued beads combined with quantum dots in a microarray. Nano-Micro Lett..

[186] Soda N, Rehm BHA, Sonar P, Nguyen NT, Shiddiky MJA (2019). Advanced liquid biopsy technologies for circulating biomarker detection. J. Mater. Chem. B.

[187] Guo Q, Wang Y, Chen C, Wei D, Fu J (2020). Multiplexed luminescence oxygen channeling immunoassay based on dual-functional barcodes with a host-guest structure: a facile and robust suspension array platform. Small.

[188] Bian F, Sun L, Cai L, Wang Y, Zhao Y (2020). Bioinspired Mxene-integrated colloidal crystal arrays for multichannel bioinformation coding. PNAS.

[189] Yang M, Liu Y, Jiang X (2019). Barcoded point-of-care bioassays. Chem. Soc. Rev..

[190] Geng Y, Peveler WJ, Rotello VM (2019). Array-based “chemical nose” sensing in diagnostics and drug discovery. Angew. Chem. Int. Ed..

[191] You CC, Miranda OR, Gider B, Ghosh PS, Kim IB (2007). Detection and identification of proteins using nanoparticle–fluorescent polymer ‘chemical nose’ sensors. Nat. Nanotechnol..

[192] Yu T, Xianyu Y (2021). Array-based biosensors for bacteria detection: from the perspective of recognition. Small.

[193] Liu H, Xiong LH, Kwok RTK, He X, Lam JWY (2020). AIE bioconjugates for biomedical applications. Adv. Opt. Mater..

[194] Yaraki MT, Wu M, Middha E, Wu W, Rezaei SD (2021). Gold nanostars-AIE theranostic nanodots with enhanced fluorescence and photosensitization towards effective image-guided photodynamic therapy. Nano-Micro Lett..

[195] Li Y, Li S, Wang J, Liu G (2019). Crispr/cas systems towards next-generation biosensing. Trends Biotechnol..

[196] Li Y, Liu L, Liu G (2019). CRISPR/Cas multiplexed biosensing: a challenge or an insurmountable obstacle?. Trends Biotechnol..

[197] L. Hao, R.T. Zhao, C. Ngambenjawong, H.E. Fleming, S.N. Bhatia, CRISPR-Cas-amplified urine biomarkers for multiplexed and portable cancer diagnostics. bioRxiv (2020). 10.1101/2020.06.17.157180 10.1038/s41565-023-01372-9 PMC10359190 37095220

[198] Dai Y, Wu Y, Liu G, Gooding JJ (2020). CRISPR mediated biosensing toward understanding cellular biology and point-of-care diagnosis. Angew. Chem. Int. Ed..

[199] Gootenberg JS, Abudayyeh OO, Kellner MJ, Joung J, Collins JJ (2018). Multiplexed and portable nucleic acid detection platform with Cas13, Cas12a, and Csm6. Science.

[200] Yue H, Shu B, Tian T, Xiong E, Huang M (2021). Droplet Cas12a assay enables DNA quantification from unamplified samples at the single-molecule level. Nano Lett..

[201] Ackerman CM, Myhrvold C, Thakku SG, Freije CA, Metsky HC (2020). Massively multiplexed nucleic acid detection with Cas13. Nature.

[202] Weckman NE, Ermann N, Gutierrez R, Chen K, Graham J (2019). Multiplexed DNA identification using site specific dCas9 barcodes and nanopore sensing. ACS Sens..

[203] Bruch R, Urban GA, Dincer C (2019). Crispr/Cas powered multiplexed biosensing. Trends Biotechnol..

[204] R. Bruch, J. Baaske, C. Chatelle, W. Weber, C. Dincer et al. Electrochemical biosensor for Crispr/Cas13a powered miRNA diagnostics. 2019 IEEE SENSORS, Montreal, QC, Canada, 27–30 Oct. (2019). https://ieeexplore.ieee.org/document/8956561

[205] Mei F, Fancy SPJ, Shen YAA, Niu J, Zhao C (2014). Micropillar arrays as a high-throughput screening platform for therapeutics in multiple sclerosis. Nat. Med..

[206] Zhang P, Wu X, Gardashova G, Yang Y, Zhang Y (2020). Molecular and functional extracellular vesicle analysis using nanopatterned microchips monitors tumor progression and metastasis. Sci. Transl. Med..

[207] Zhang P, Zhou X, He M, Shang Y, Tetlow AL (2019). Ultrasensitive detection of circulating exosomes with a 3D-nanopatterned microfluidic chip. Nat. Biomed. Eng..

[208] Huang L, Tian S, Zhao W, Liu K, Ma X (2020). Multiplexed detection of biomarkers in lateral-flow immunoassays. Analyst.

[209] Zhao W, Tian S, Huang L, Liu K, Dong L (2020). A smartphone-based biomedical sensory system. Analyst.

[210] Dincer C, Bruch R, Kling A, Dittrich PS, Urban GA (2017). Multiplexed point-of-care testing–Xpoct. Trends Biotechnol..

[211] Kaur J, Jiang C, Liu G (2019). Different strategies for detection of HBA1C emphasizing on biosensors and point-of-care analyzers. Biosens. Bioelectron..

[212] Dong Z, Tang C, Zhang Z, Zhou W, Zhao R (2020). Simultaneous detection of exosomal membrane protein and RNA by highly sensitive aptamer assisted multiplex–PCR. ACS Appl. Bio Mater..

[213] Lim CZJ, Natalia A, Sundah NR, Shao H (2020). Biomarker organization in circulating extracellular vesicles: new applications in detecting neurodegenerative diseases. Adv. Biosyst..

[214] K.E. Richards, D.B. Go, R. Hill (2017) Surface acoustic wave lysis and ion-exchange membrane quantification of exosomal microRNA. *MicroRNA Detection and Target Identification. Methods in Molecular Biology* chapter 5, 59–70. 10.1007/978-1-4939-6866-4_5 10.1007/978-1-4939-6866-4_5 28439826

[215] Taller D, Richards K, Slouka Z, Senapati S, Hill R (2015). On-chip surface acoustic wave lysis and ion-exchange nanomembrane detection of exosomal RNA for pancreatic cancer study and diagnosis. Lab Chip.

[216] Zhu F, Ji Y, Deng J, Li L, Bai X (2021). Microfluidics-based technologies for the analysis of extracellular vesicles at the single-cell level and single-vesicle level. Chin. Chem. Lett..

[217] Orooji Y, Sohrabi H, Hemmat N, Oroojalian F, Baradaran B (2020). An overview on SARS-Cov-2 (COVID-19) and other human coronaviruses and their detection capability via amplification assay, chemical sensing, biosensing, immunosensing, and clinical assays. Nano-Micro Lett..

[218] Cao C, Zhang Y, Jiang C, Qi M, Liu G (2017). Advances on aryldiazonium salt chemistry based interfacial fabrication for sensing applications. ACS Appl. Mater. Interfaces.

[219] Krug A, Enderle D, Karlovich C, Priewasser T, Bentink S (2018). Improved EGFR mutation detection using combined exosomal RNA and circulating tumor DNA in NSCLC patient plasma. Ann. Oncol..

[220] Shin H, Oh S, Hong S, Kang M, Kang D (2020). Early-stage lung cancer diagnosis by deep learning-based spectroscopic analysis of circulating exosomes. ACS Nano.

[221] Riordon J, Sovilj D, Sanner S, Sinton D, Young EW (2019). Deep learning with microfluidics for biotechnology. Trends Biotechnol..

[222] Min L, Wang B, Bao H, Li X, Zhao L (2021). Advanced nanotechnologies for extracellular vesicle-based liquid biopsy. Adv. Sci..

[223] Fraser K, Jo A, Giedt J, Vinegoni C, Yang KS (2019). Characterization of single microvesicles in plasma from glioblastoma patients. Neuro Oncol..

